# Inhibition of the *Staphylococcus aureus* c-di-AMP cyclase DacA by direct interaction with the phosphoglucosamine mutase GlmM

**DOI:** 10.1371/journal.ppat.1007537

**Published:** 2019-01-22

**Authors:** Tommaso Tosi, Fumiya Hoshiga, Charlotte Millership, Rahul Singh, Charles Eldrid, Delphine Patin, Dominique Mengin-Lecreulx, Konstantinos Thalassinos, Paul Freemont, Angelika Gründling

**Affiliations:** 1 Section of Microbiology and MRC Centre for Molecular Bacteriology and Infection, Imperial College London, London, United Kingdom; 2 Institute of Structural and Molecular Biology, Birkbeck College, University of London, Malet Street, London, United Kingdom; 3 Institute of Structural and Molecular Biology, Division of Biosciences, University College London, London, United Kingdom; 4 Institute for Integrative Biology of the Cell, CEA, CNRS, Univ Paris-Sud and Université Paris-Saclay, Gif-sur-Yvette, France; 5 Section of Structural Biology, Department of Medicine, Imperial College London, London, United Kingdom; National Institutes of Health, UNITED STATES

## Abstract

c-di-AMP is an important second messenger molecule that plays a pivotal role in regulating fundamental cellular processes, including osmotic and cell wall homeostasis in many Gram-positive organisms. In the opportunistic human pathogen *Staphylococcus aureus*, c-di-AMP is produced by the membrane-anchored DacA enzyme. Inactivation of this enzyme leads to a growth arrest under standard laboratory growth conditions and a re-sensitization of methicillin-resistant *S*. *aureus* (MRSA) strains to ß-lactam antibiotics. The gene coding for DacA is part of the conserved three-gene *dacA/ybbR*/*glmM* operon that also encodes the proposed DacA regulator YbbR and the essential phosphoglucosamine mutase GlmM, which is required for the production of glucosamine-1-phosphate, an early intermediate of peptidoglycan synthesis. These three proteins are thought to form a complex *in vivo* and, in this manner, help to fine-tune the cellular c-di-AMP levels. To further characterize this important regulatory complex, we conducted a comprehensive structural and functional analysis of the *S*. *aureus* DacA and GlmM enzymes by determining the structures of the *S*. *aureus* GlmM enzyme and the catalytic domain of DacA. Both proteins were found to be dimers in solution as well as in the crystal structures. Further site-directed mutagenesis, structural and enzymatic studies showed that multiple DacA dimers need to interact for enzymatic activity. We also show that DacA and GlmM form a stable complex *in vitro* and that *S*. *aureus* GlmM, but not *Escherichia coli* or *Pseudomonas aeruginosa* GlmM, acts as a strong inhibitor of DacA function without the requirement of any additional cellular factor. Based on Small Angle X-ray Scattering (SAXS) data, a model of the complex revealed that GlmM likely inhibits DacA by masking the active site of the cyclase and preventing higher oligomer formation. Together these results provide an important mechanistic insight into how c-di-AMP production can be regulated in the cell.

## Author summary

c-di-AMP has recently emerged as an essential molecule in Gram-positive bacteria, controlling osmotic stress resistance and virulence. The molecule has also been linked to antibiotic resistance/sensitivity, as alterations in its levels have been shown to modulate the effect of cell wall-targeting antibiotics. c-di-AMP is produced and degraded by dedicated enzymes and the activities of these enzymes are tightly regulated in the cell for optimal bacterial growth. In the human pathogen *Staphylococcus aureus*, c-di-AMP is produced by the di-adenylate cyclase enzyme DacA, which is thought to form a complex and be regulated by YbbR and GlmM, two other conserved proteins. There are ongoing efforts to devise strategies to inhibit di-adenylate cyclase enzymes, as inhibition of c-di-AMP production will negatively impact bacterial growth and also re-sensitize methicillin-resistant *S*. *aureus* (MRSA) strains to ß-lactam antibiotics. Here, we investigated the activity and regulation of the *S*. *aureus* c-di-AMP cyclase DacA. We determined the atomic structures of the *S*. *aureus* DacA and GlmM enzymes and show that GlmM readily binds to DacA *in vitro* and blocks its activity by masking its active site. With this, our work provides important insight into possible ways to ablate c-di-AMP production in bacterial cells.

## Introduction

For pathogenic bacteria, the ability to rapidly adapt to the host cell environment or different host cell niches is essential for their infectivity. Nucleotide second-messenger molecules are critical components involved in such adaptive responses, allowing bacteria to simultaneously regulate multiple cellular processes [1]. c-di-AMP has emerged as an important second-messenger, in particular in Gram-positive bacteria [2, 3]. Several recent studies have shown that c-di-AMP binds to and regulates cellular transport systems for potassium and osmolytes and in this manner likely controls the cellular turgor [2, 4–9]. Therefore c-di-AMP has an important role in preserving the integrity and viability of the cell when osmotic conditions change. Furthermore, in a number of Gram-positive bacteria, including the human pathogens *Staphylococcus aureus* and *Listeria monocytogenes*, c-di-AMP impacts the susceptibility to ß-lactam antibiotics [10–14]. More specifically, high cellular c-di-AMP levels lead to increased ß-lactam resistance and low cellular c-di-AMP levels to decreased resistance [12–15]. Although the molecular mechanism behind this is still unclear, it has been suggested that c-di-AMP might at least in part impact ß-lactam resistance through its regulatory function of potassium and osmolyte transporters and changes in osmotic balance and pressure [9, 16]. These findings indicate that blocking c-di-AMP production could potentially be used to re-sensitize pathogens such as methicillin-resistant *S*. *aureus* (MRSA) strains to ß-lactam antibiotics. In order to exploit such strategies, a better knowledge of the structure and function of c-di-AMP synthesis enzymes and the modulation of their activity is required.

For optimal fitness, bacteria need to tightly regulate c-di-AMP production [8, 17–19] [6, 11, 20–22]. Its cellular levels are regulated by the balance between its synthesis by dedicated di-adenylate cyclase enzymes (DacA in *S*. *aureus*) and its degradation to pApA or AMP by specific phosphodiesterase enzymes [12, 23–28]. These enzymes are part of three main classes, represented by DisA, DacA (CdaA) and CdaS [23, 24, 29]. Structural and biochemical characterization of members of the different c-di-AMP cyclase families have revealed important information on their regulation. The first di-adenylate cyclase to be characterized was the DNA integrity scanning protein DisA from *Thermotoga maritima* [23]. DisA is a modular cytoplasmic protein, in which the di-adenylate cyclase domain (from here on referred to as DAC domain) is linked by a helical linker region to a DNA-binding domain. The protein was present as an octamer (tetramer of dimers) in the crystal structure, with the DAC domains in a head-to-head conformation [23, 30]. DisA dependent c-di-AMP production is regulated through multiple mechanisms. Both, the interaction of DisA with the DNA repair protein RadA and the binding of the DisA-associated DNA-binding domain to damaged DNA, negatively impact its activity [23, 31, 32].

Besides DisA, the structure of the CdaS cyclase from *Bacillus cereus* is available (PDB 2FB5) and the homologous proteins from *Bacillus subtilis* and *Bacillus thuringiensis* have been characterized biochemically [33, 34]. The cyclase domain in CdaS proteins is preceded by an N-terminal regulatory YojJ domain that is composed of two long alpha-helices. The structural and biochemical analyses of CdaS proteins from different *Bacillus* species indicate that the protein is a hexamer in the crystal structure as well as in solution [33, 34]. However, in contrast to the oligomeric DisA protein, the *B*. *subtilis* CdaS enzyme seemed to be only weakly active in this higher oligomeric form, as the DAC domains were not found in a head-to-head conformation [33]. Deletion of the N-terminal regulatory helices of the *B*. *subtilis* CdaS protein disrupted hexamer formation, yielding protein dimers with high enzymatic activity [33]. In contrast, deletion of the N-terminal regulatory domain in the *B*. *thuringiensis* CdaS protein greatly inhibited the enzymatic activity of this enzyme. Therefore, while it is clear that the c-di-AMP activity of CdaS-type proteins is regulated by the N-terminal YojJ domain, the exact regulatory mechanism is still unclear and might also differ between different *Bacillus* spp.

CdaA-type (DacA-type) enzymes are widely distributed among bacteria. As many important Gram-positive pathogens produce this type of cyclase, inhibiting the function of members of this class of enzyme will most likely have the biggest therapeutic potential. The soluble enzymatic domain of the membrane-bound CdaA enzyme from *L*. *monocytogenes* has been crystallized [35]. While the CdaA enzyme from *L*. *monocytogenes* was present as a crystallographic tetramer in the structure, none of the subunits were engaged in a catalytically competent head-to-head conformation.

The *cdaA* genes (including *dacA* in *S*. *aureus*) encoded by Firmicutes are located in a highly conserved three- or four-gene operon that includes the conserved genes *ybbR* and *glmM* [17, 18], revealing a potentially interesting connection to cell wall biosynthesis. GlmM codes for the essential phosphoglucosamine mutase enzyme, required for the conversion of glucosamine-6-phosphate to the early peptidoglycan synthesis precursor glucosamine-1-phosphate [36, 37]. Recent work has highlighted a direct interaction between CdaA, YbbR and GlmM [17, 18]. Bacterial two-hybrid (B2H) assays performed with the *B*. *subtilis* or *Lactococcus lactis* proteins indicated that YbbR interacts with the transmembrane region of CdaA, whilst GlmM interacts with the cytoplasmic catalytic domain of CdaA [17, 38]. Further work in *L*. *lactis* indicated that the interaction of GlmM with CdaA leads to a decrease in the cellular levels of c-di-AMP, suggesting an inhibitory role of GlmM on the CdaA c-di-AMP cyclase enzyme [17]. However, biochemical and atomic level details of the GlmM/CdaA (DacA) interaction are currently lacking.

As part of this study, we investigated the regulatory mechanism of the *S*. *aureus* c-di-AMP cyclase DacA, including the interaction of GlmM with DacA, structurally and biochemically. We determined the structures of the two essential *S*. *aureus* enzymes, GlmM and the enzymatic domain of DacA and also showed that these proteins form a stable complex *in vitro* without the requirement of any additional factors. Upon binding, GlmM completely abolished the activity of the DacA cyclase enzyme, while GlmM activity was not significantly affected upon complex formation. SAXS envelope data of the purified DacA/GlmM complex allowed us to propose a molecular model for how GlmM inhibits the activity of the c-di-AMP cyclase by blocking access to its catalytic site. Thus, our study provides important new insight into how c-di-AMP production can be blocked, paving the way to devising new strategies to interfere with c-di-AMP production in important human pathogens such as *S*. *aureus*.

## Results

### Apo-structure of the *S*. *aureus* c-di-AMP cyclase DacA_CD_

**Fig 1:**
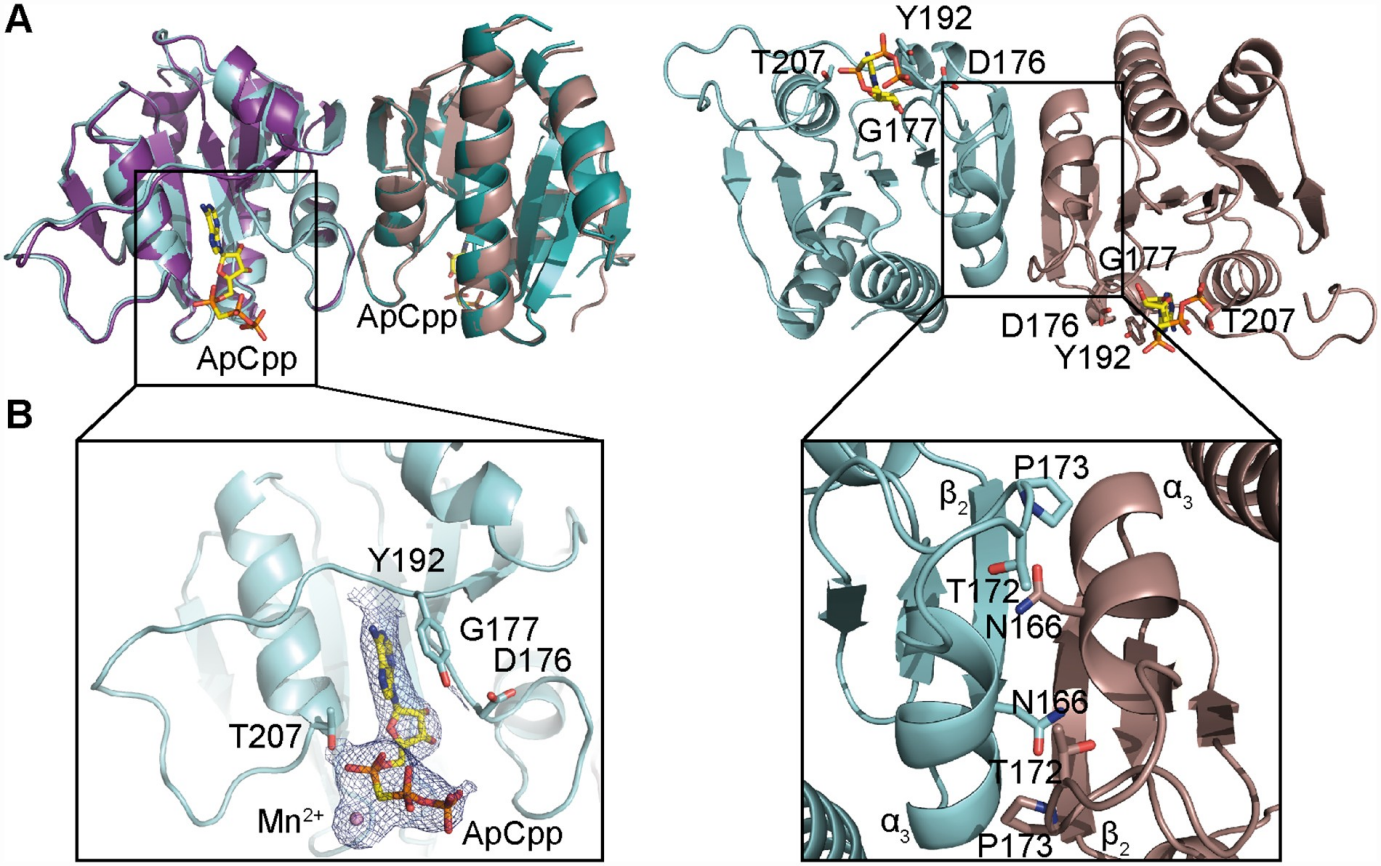
Crystal structure of the *S*. *aureus* DacA_CD_ protein in the nucleotide-free and nucleotide-bound states. (A) Protein structure of the *S*. *aureus* DacA_CD_ protein with and without ApCpp, shown in ribbon representation. The apo *S*. *aureus* DacA_CD_ structure was solved by molecular replacement using the *L*. *monocytogenes* CdaA protein (PDB 4RV7) as template. The two monomers in the crystallographic unit are shown in teal and purple (for apo-DacA_CD_) and in cyan and brown (for ApCpp-DacA_CD_). The dimer is shown in two views with the second view in a 90° angle with the predicted active site residues D176, G177, T207 shown in stick representation in the 90° view (right). While the *dacA* sequence starting from codon F101 were included in the construct, only residues Y110 to G260 were visible in chain A and residues S111 to T261 in chain B. (B) Zoomed-in view of the *S*. *aureus* ApCpp-DacA_CD_ binding site (left) and DacA_CD_ dimerization interface (right). Electron densities corresponding to ApCpp and the metal ion are displayed. ApCpp and the DacA_CD_ interacting residues are shown in stick representation. Amino acid residues N166, T172 and P173 providing hydrogen bonding or van der Waals interactions are also shown in stick representation.

The *S*. *aureus* c-di-AMP cyclase enzyme DacA has three N-terminal transmembrane helices, which are followed by the cytoplasmically located enzymatic DisA_N domain. To gain further insight into the function and catalytic mechanism of the *S*. *aureus* c-di-AMP cyclase DacA, the soluble catalytic domain starting from amino acid F101 (hereafter referred to as DacA_CD_) was expressed and purified as N-terminal His-tag fusion protein. Following removal of the His-tag, the protein was crystallized and the structure of the nucleotide-free DacA_CD_ domain was obtained by molecular replacement using the *L*. *monocytogenes* CdaA protein (PDB 4RV7) as a search model [35] (Fig 1 and S1 Table). The two proteins have an identify of 51.7% and the structures overlapped with a root-mean-square deviation (RMSD) of 0.438 Å. The *S*. *aureus* DacA_CD_ protein displayed the expected globular fold, with a central β-sheet made up of 7 antiparallel strands flanked by 5 helices (Fig 1A, left). While the *L*. *monocytogenes* CdaA_CD_ protein was found to be a tetramer in the asymmetric unit (although dimeric in solution) [35], the *S*. *aureus* DacA_CD_ was present as a symmetric dimer, with two protomers interacting via the backbone of α3, β2 and the loop connecting α3 and β3 (Fig 1A and 1B). At the interface, residue N166 from one protomer formed a hydrogen bond interaction with residue T172 from the second protomer and a van der Waals interaction was formed between residue P173 and residues N166 and I165 of the neighbouring protomer (Fig 1B). Further analysis using the PdbePISA program [39] revealed a total buried surface area of 1450 Å^2^ at the dimer interface with a ΔG (int) of -8.3 kcal/mol and a ΔG (diss) of 2.1 kcal/mol, indicative of a strong protein/protein interaction.

### Structure of *S*. *aureus* DacA_CD_ bound to ApCpp

In order to condense two ATP molecules to c-di-AMP, two protomers need to be arranged in a head-to-head conformation [23, 35]. However, in the *S*. *aureus* DacA_CD_ dimer found in the crystallographic asymmetric unit, the active sites of the protomers are outward facing and hence are too far apart for c-di-AMP synthesis (Fig 1A). In addition, analysis of the crystal lattice did not reveal any symmetry-related molecule arranged with the catalytic sites in a head-to-head orientation. To determine whether nucleotide binding could trigger a conformational change of the protein leading to inter-protomer rearrangement, the *S*. *aureus* DacA_CD_ protein was crystallized in the presence of ApCpp, a non-hydrolyzable ATP substrate as well as 2 mM MnCl_2_. Crystals were readily obtained and diffracted to 2.7Å (Fig 1 and S1 Table). The structure was solved by molecular replacement using the structure of the nucleotide-free *S*. *aureus* DacA_CD_ domain as a search model. The DacA_CD_ protein co-crystallized with ApCpp had the same dimeric arrangement as observed for the nucleotide-free protein, but extra densities for the ApCpp nucleotide were observed in each protomer (Fig 1). As expected, the ApCpp molecule was found in the catalytic pocket with residues D176 and G177 interacting with the ribose moiety, residue T207 interacting with the phosphate backbone and residue Y192 engaged in a π-stacking interaction with the adenine base (Fig 1B, left). The overall protein structures of the nucleotide-free and the nucleotide-bound proteins were very similar and overlapped with an RMSD of 0.34 Å (Fig 1A). Our data suggest that nucleotide binding does not trigger any major conformational changes leading to a rearrangement of the DacA_CD_ dimer. However, we did observe a near head-to-head configuration of DacA_CD_ by inspecting neighbouring molecules within the crystal lattice (S1A Fig). While the individual protomers in neighbouring units are too far apart to be engaged in a catalytic reaction, the data do suggest that the protein can form a catalytically active dimeric conformation by forming higher-order oligomers. Furthermore, overlaying the DacA_CD_ -ApCpp structure with a dimer of the *B*. *subtilis* DisA structure highlighted that there is sufficient space for two *S*. *aureus* DacA_CD_ dimers to interact in a head-to-head orientation as required for catalysis (S1B Fig).

### Enzymatic activity and characterization of the *S*. *aureus* DacA_CD_ dimer interface

**Fig 2:**
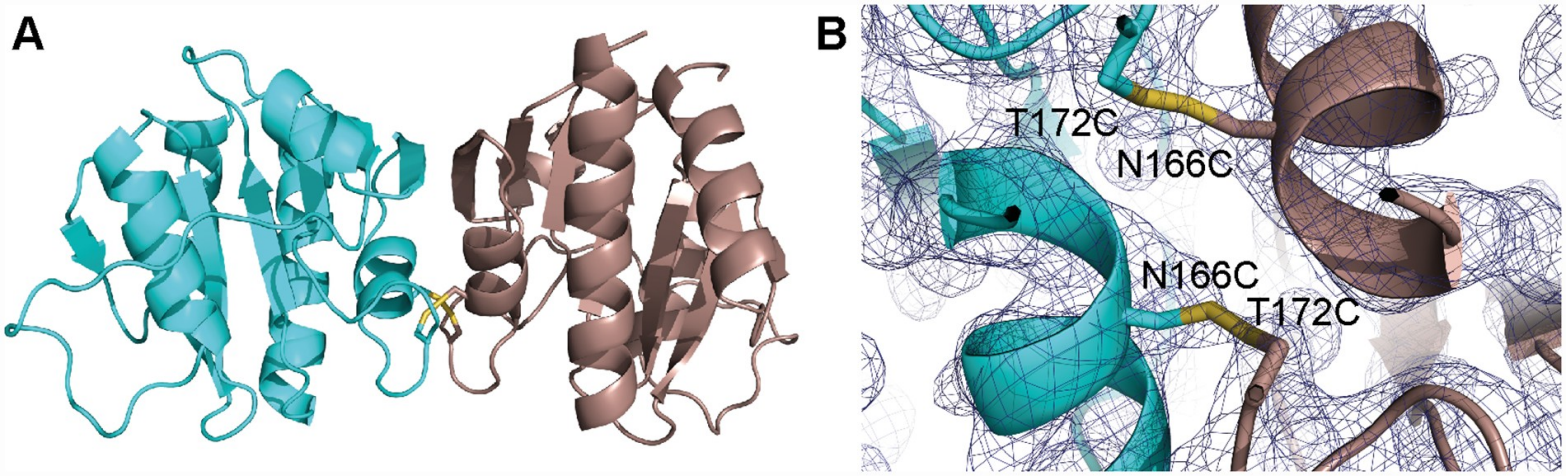
Structure of the DacA_CD_-C variant. (A) X-ray structure of the DacA_CD_-C variant. The structure of the tag-less DacA_CD_-C variant was solved by molecular replacement using the WT *S*. *aureus* DacA_CD_ protein shown in Fig 1 as template. The DacA_CD_-C protein was found as a dimer in the crystallographic unit. (B) Electron density of the dimer interface in DacA_CD_-C, showing electron densities consistent with the formation of two disulfide bonds between two DacA_CD_-C monomers at sigma 1.3.

To determine whether the observed DacA_CD_ dimer could be physiologically relevant and enzymatically active, we next performed enzyme activity assays using different DacA_CD_ variants to test the relevance of the observed dimer interface. Two key interacting residues in the dimer interface are amino acids N166 and T172 (Fig 1B). To potentially disrupt this interaction, we mutated these two residues to lysines, yielding variant DacA_CD_-K. To “lock” the dimer conformation, we also mutated these residues to cysteines (DacA_CD_-C variant) to facilitate the formation of disulfide bonds between the two protomers. WT DacA_CD_ and the DacA_CD_-K and DacA_CD_-C variants were purified and the oligomeric state of the proteins was estimated by size exclusion chromatography. The WT DacA_CD_ and DacA_CD_-C variant had similar elution profiles and eluted at a retention volume as expected for a DacA_CD_ dimer (S2 Fig). The main peak for the DacA_CD_-K variant eluted with the same retention volume as the WT DacA_CD_ and DacA_CD_-C variants indicating that the protein was still able to form a dimer. However, additional faster and slower eluting peaks were observed for the DacA_CD_-K variant indicative of protein unfolding or aggregation (earlier eluting peak) and monomer formation (later eluting peak) (S2 Fig). These data indicate that introduction of lysine residues partially disrupts DacA_CD_ dimer formation. To further assess if the DacA_CD_-C variant could form disulfide-bridge locked dimers, the purified protein was crystallized and its structure determined. The protein crystallized in the same dimeric conformation as the DacA_CD_ protein and electron densities consistent with the formation of two disulfide bonds were observed for the DacA_CD_-C variant, which were absent in the DacA_CD_ protein (Fig 2 and S1 Table).

**Fig 3:**
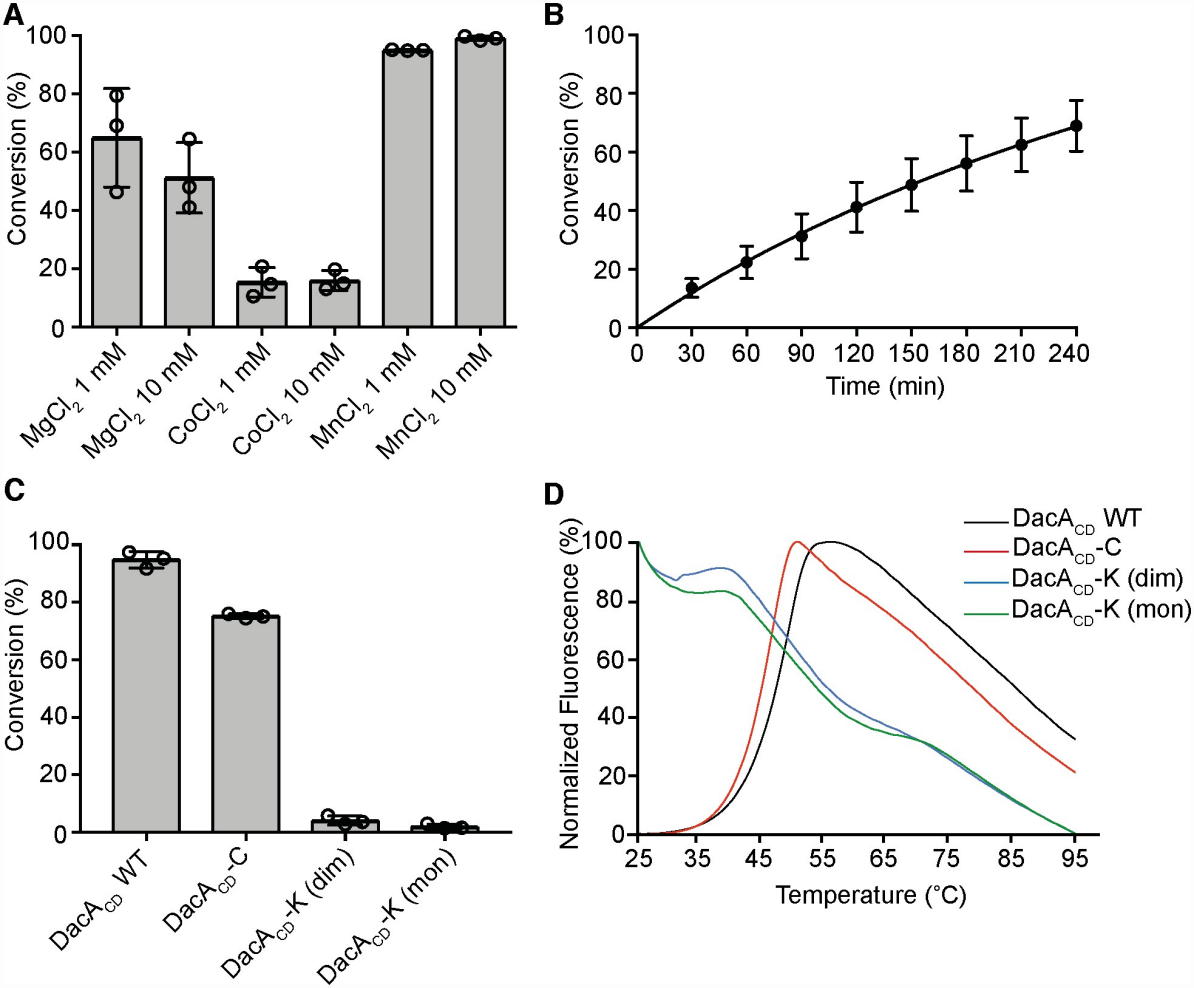
Activity assays of DacA_CD_ and its variants. (A) Enzyme activity assay and metal-dependency of the WT DacA_CD_ protein. 5 μM DacA_CD_ was incubated with 100 μM M ATP spiked with a small amount of radiolabeled ATP in buffer containing 1 mM or 10 mM of Mg^2+^, Co^2+^ or Mn^2+^ metal ions. The reactions were incubated for 4 h at 37°C and % ATP to c-di-AMP conversion determined. The average values and standard deviations from three independent experiments were plotted. (B) DacA_CD_ cyclase activity time-course experiment. 5 μM DacA_CD_ was incubated with 100 μM ATP spiked with radiolabeled ATP in buffer containing 10 mM Mn^2+^ and the reactions were stopped at the indicated time points. The % ATP that was converted to c-di-AMP was determined and the average values and standard deviations from three independent experiments plotted. (C) Cyclase activity of WT DacA_CD_, DacA_CD_-C and DacA_CD_-K variants. The indicated protein was incubated with ATP for 4 h as described in panel B and the average values of % ATP conversion and standard deviations from three independent experiments were plotted. (D) Thermofluor melting curves. 5 μM of the indicated protein was incubated with SYPRO Orange dye, the solution was heated from 25 to 95°C in 1°C increments and the fluorescence readings determined. The melting curve for WT DacA_CD_ is shown in black, for the DacA_CD_-C variant in red, for the DacA_CD_-K variant derived from the presumed dimer peak in blue and the DacA_CD_-K variant derived from the presumed monomer peak in green.

Next, the purified proteins were tested for enzyme activity by quantifying the conversion of α-P^32^-labelled ATP into P^32^-labelled c-di-AMP. First, the metal dependency of the WT DacA_CD_ protein was assessed. To this end, DacA_CD_ was incubated with ATP spiked with a small amount of α-P^32^-labelled ATP in buffers containing 1 or 10 mM of one of the divalent cations Mg^2+^, Co^2+^ or Mn^2+^. After 4 h the reactions were stopped and the input ATP and c-di-AMP reaction product quantified by phosphorimaging. This analysis revealed that the *S*. *aureus* DacA_CD_ protein is most active in the presence of Mn^2+^ and hence all subsequent experiments were performed in the presence of 10 mM MnCl_2_ (Fig 3A). Next, a time course experiment was performed and the DacA_CD_ enzyme displayed an *in vitro* catalytic activity with an average catalytic rate of 2.28x10^-10^ M/min (Fig 3B). This activity is slow, but consistent with previous observations [23]. To assess the activity of the different DacA_CD_ variants, enzyme activity assays were performed with the DacA_CD_, DacA_CD_-C, or DacA_CD_-K (either obtained from the predicted monomer or dimer peak) proteins. The DacA_CD_-C variant displayed very similar activity to the WT DacA_CD_ protein (Fig 3C), suggesting that locking the dimer through two disulfide bridges does not influence the activity of the enzyme. In contrast, the DacA_CD_-K variant derived either from the late eluting (predicted monomer) or earlier eluting (predicted dimer) peaks displayed very low activity (Fig 3C). We reasoned that the activity loss could be due to an intrinsic instability of this protein variant. To test this, the stability of the DacA_CD_-K variant was assessed using a thermofluor assay and compared to that of the WT DacA_CD_ and DacA_CD_-C variant. The melting curves for the DacA_CD_ and DacA_CD_-C proteins were sigmoidal, which is typical for well-folded proteins (Fig 3D). On the other hand, the DacA_CD_-K variant displayed high levels of fluorescence from the beginning of the melting curve (Fig 3D), which is indicative of an unfolded protein and likely explains the low enzymatic activity of the DacA_CD_-K variant. Taken together, these data show that WT DacA_CD_ and the ‘locked-dimer’ DacA_CD_-C variant show similar enzymatic activities, suggesting that the stable dimer we observed in our crystal structures will likely transiently interact with other dimers to form higher oligomers for catalysis and c-di-AMP product formation.

### *S*. *aureus* DacA_CD_ and GlmM form a complex *in vitro*

**Fig 4:**
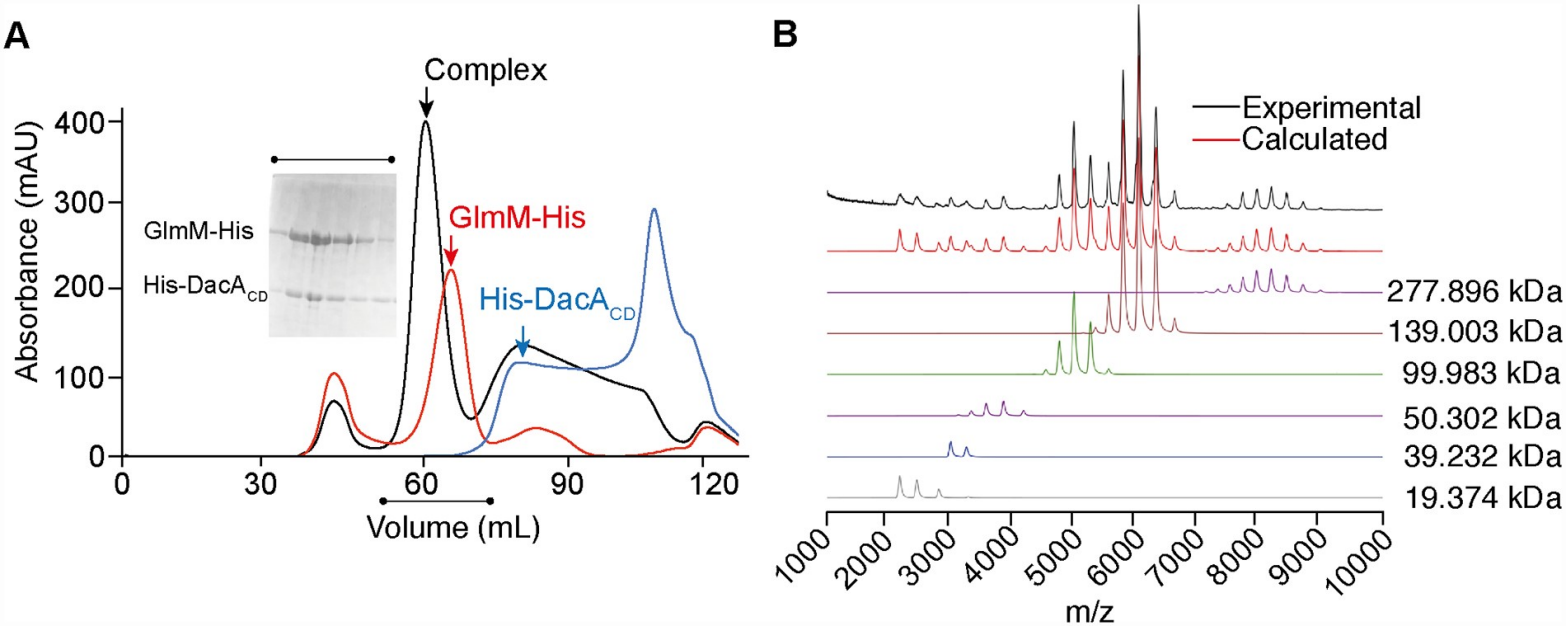
The *S*. *aureus* DacA_CD_ and GlmM proteins form a stable complex *in vitro*. (A) Size exclusion profiles of the His-DacA_CD_ (blue line), GlmM-His (red line) proteins and the His-DacA_CD_/GlmM-His complex (black line). The insert shows a Coomassie-stained gel with aliquots of fractions from the complex peak. The experiment was performed three times and a representative result is shown. (B) Native mass-spectrometry experiment of the DacA/GlmM complex. The spectrum was deconvoluted using the software Amphitrite [53]. Experimental and calculated spectra are shown in black and red, respectively. Charge state distributions corresponding to the different detected species are depicted in different colours. The molecular weights reported correspond to: DacA/GlmM dimer complex (purple line, 277.896 kDa, four copies of DacA_CD_ with four copies of GlmM); DacA/GlmM complex (brown line, 139.003 kDa, two copies of DacA_CD_ with two copies of GlmM–main DacA/GlmM complex); GlmM dimer (green line, 99.983 kDa); GlmM monomer (purple line—50.302 kDa); DacA_CD_ dimer (blue line—39.232 kDa); DacA_CD_ monomer (grey line—19.374 kDa) (S2 Table).

Results from *in vivo* crosslinking and bacterial two-hybrid experiments suggested that the phosphoglucosamine mutase GlmM can interact with the DacA homologs in *B*. *subtilis* and *L*. *lactis* [17, 18]. To assess whether the *S*. *aureus* DacA_CD_ protein can interact with the *S*. *aureus* GlmM protein, a plasmid for the overexpression of a C-terminally His-tagged *S*. *aureus* GlmM protein (GlmM-His) was generated. Next, expression of the His-DacA_CD_ and GlmM-His proteins was induced in *E*. *coli*, the cells lysed and the proteins purified by Ni-affinity and size exclusion chromatography, or *E*. *coli* lysates from His-DacA_CD_ and GlmM-His overexpressing strains mixed before affinity and size exclusion chromatography. When the two proteins were purified together, a new faster eluting peak corresponding to a species of higher molecular weight and indicating the formation of a complex, was obtained (Fig 4A). SDS-PAGE analysis of aliquots from the different elution fractions confirmed the co-elution of the His-DacA_CD_ and GlmM-His proteins in the high molecular weight peak (Fig 4A). To further confirm the interaction of DacA_CD_ with GlmM *in vitro*, a pull-down assay was performed. For this purpose, the His-tag was removed from DacA_CD_ and the purified tag-less protein subsequently mixed in equimolar amount with purified GlmM-His. The protein mixture was then applied to a Ni-NTA column and after two wash steps, bound proteins were eluted. The untagged DacA_CD_ protein quantitatively co-eluted with GlmM-His, confirming the protein/protein interaction (S3 Fig). As control, the tag-less DacA_CD_ protein was subjected to the same procedure in the absence of GlmM-His. In this case, the protein was found in the load and wash fractions. Taken together, these results show that DacA and GlmM can form a stable complex *in vitro*, and that the two proteins can be co-purified as a single species. To estimate the size and stoichiometry of the complex, the purified DacA_CD_-GlmM complex was further analyzed by SEC-MALS as well as native mass spectrometry. Based on the SEC-MALS elution profile, the complex had an estimated molecular weight of 129 kDa ± 0.4% (S4 Fig) and based on the native mass spectrometry analysis, the major species in the spectrum corresponded to a complex of 139.003 kDa ± 10.41 Da (Fig 4B). These data are consistent with a DacA_CD_-GlmM complex comprising one GlmM dimer (theoretical mass 99.462 kDa) interacting with one DacA_CD_ dimer (theoretical mass 19.374 kDa), with a theoretical molecular weight of 138.21 kDa (S2 Table).

### *S*. *aureus* GlmM is a negative regulator of DacA activity both *in vitro* and *in vivo*

**Fig 5:**
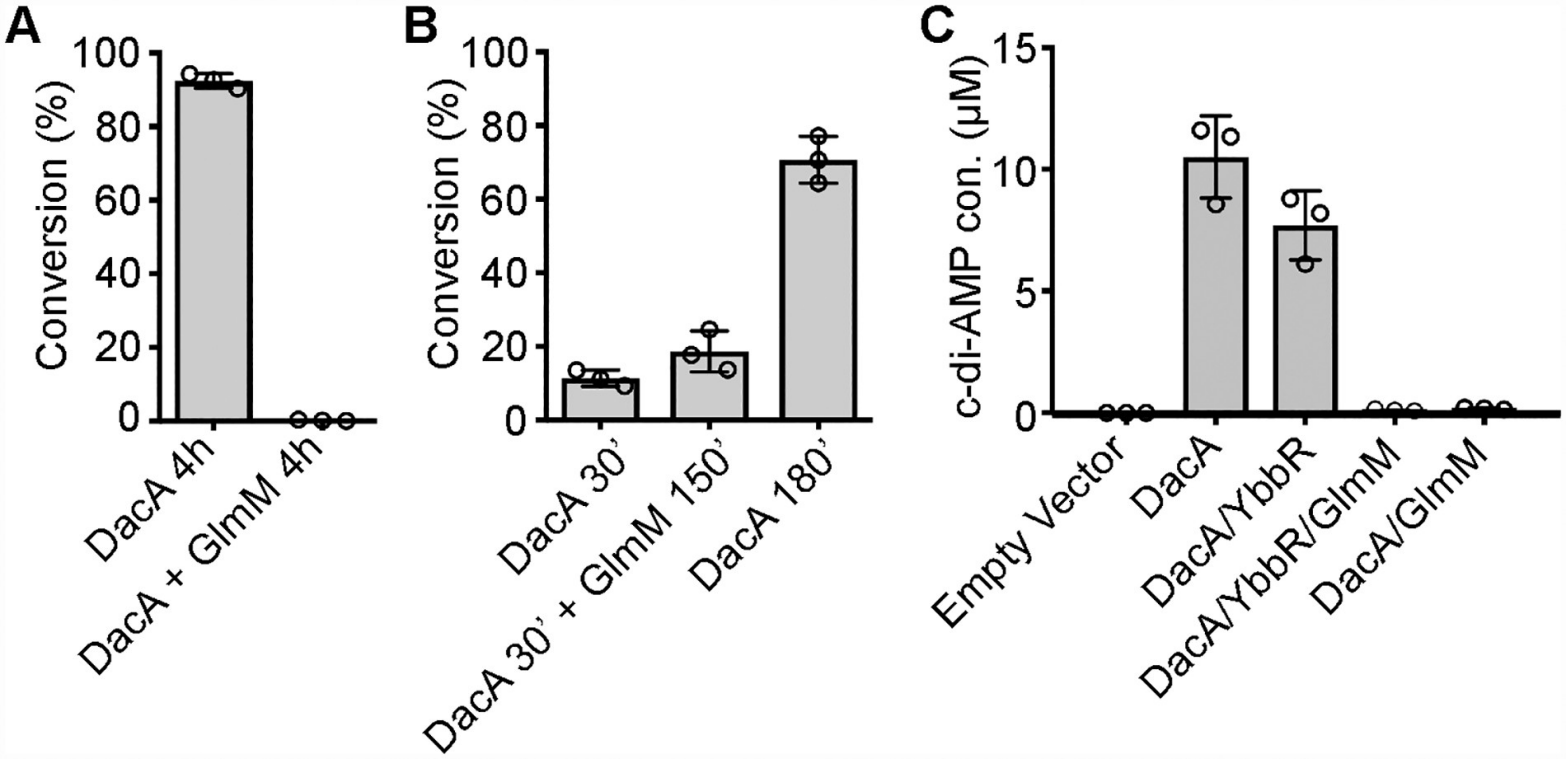
*S*. *aureus* GlmM negatively impacts the activity of DacA_CD_ *in vitro* and *in vivo*. (A) DacA_CD_ enzyme activity in the presence of GlmM. Enzyme assays were set up as described in Fig 3 in the absence or presence of 10 μM GlmM added at the start of the reactions and reactions stopped after 4 h incubation. (B) Enzyme assays were set up with 5 μM DacA_CD_ and 10 μM GlmM added after 30 min incubation and the reactions incubated for further 150 min (180 min total). As a control, DacA_CD_ enzyme reactions were also set up in the absence of GlmM and incubated for 30 or 180 min. The average values of % ATP to c-di-AMP conversion and standard deviations from three independent experiments are plotted. (C) ELISA determination of c-di-AMP levels in *E*. *coli*. Cell extracts were prepared from *E*. *coli* strains containing the empty pBAD33 vector or producing DacA, DacA/YbbR, DacA/YbbR/GlmM or DacA/GlmM. The cellular c-di-AMP levels were determined by ELISA and the average values (μM c-di-AMP per ml *E*. *coli* culture with an A_600_ of 10) and standard deviations of three independent experiments plotted.

A study with *L*. *lactis* indicated that GlmM might inhibit the activity of DacA upon binding [17]. To investigate this further, c-di-AMP production by the *S*. *aureus* DacA_CD_ enzyme was assessed in the absence or presence of GlmM. DacA_CD_ was incubated alone or mixed at a 1:2 molar ratio with GlmM, and conversion of ATP to c-di-AMP assessed after 4 h incubation at 37°C. No ATP conversion was observed in the presence of GlmM (Fig 5A). In a second experiment, a 2-fold molar excess of GlmM was added to a DacA_CD_ reaction, 30 min after initiating the reaction, and the sample subsequently incubated for a further 150 min (180 min total reaction time). As controls, 30 and 180 min DacA_CD_ enzyme reactions were also set up in the absence of GlmM. When GlmM was added 30 min following the initiation of the DacA_CD_ enzymatic cycle, the production of c-di-AMP was arrested (Fig 5B). Taken together, these data show that GlmM can effectively block the activity of the DacA_CD_ cyclase domain *in vitro* without the need for any additional cofactors.

To investigate this further and confirm that GlmM can also inhibit the activity of the full-length membrane-anchored DacA enzyme, full-length DacA was expressed in *E*. *coli* either alone, in the presence of YbbR or GlmM or in the presence of both, GlmM and YbbR, and c-di-AMP production assessed by ELISA. To this end, *dacA*, *dacA-ybbR* or the complete *dacA-ybbR-glmM* operon was cloned into plasmid pBAD33, allowing for arabinose inducible DacA, DacA/YbbR or DacA/YbbR/GlmM expression. For expression of DacA and GlmM only, the YbbR start codon was mutated and the *dacA-no-ybbR-glmM* operon inserted into plasmid pBAD33. Next, DacA and YbbR production from the different constructs was confirmed by western blot using protein-specific antibodies and GlmM production was assessed by visualizing proteins by Coomassie staining. With the exception of the empty vector control strain, similar DacA amounts were produced in all strains regardless whether or not YbbR, GlmM or YbbR and GlmM were co-expressed with the cyclase (S5 Fig). A clearly visible Coomassie stainable band of the expected size for GlmM was observed for the two strains overproducing GlmM (S5 Fig). YbbR production was also observed in the *E*. *coli* strain containing plasmids pBAD33-*dacA-ybbR* or pBAD33-*dacA-ybbR-glmM*. Mutating the YbbR start codon drastically reduced the production of YbbR but did not completely abolish its production, indicating that a second internal start codon might be utilized (S5 Fig). Next, the c-di-AMP production was assessed in the different strains. As expected, no c-di-AMP could be detected in *E*. *coli* containing the empty vector (Fig 5C). High amounts of c-di-AMP were detected upon DacA expression or expression of DacA/YbbR. On the other hand, c-di-AMP levels were drastically reduced upon co-expression of GlmM, which is consistent with the data reported by Zhu *et al*. [17] (Fig 5C). Taken together, these data show that GlmM is a negative regulator of the *S*. *aureus* c-di-AMP cyclase DacA both *in vivo* and *in vitro*. Of note, when GlmM activity was assessed *in vitro* in the presence of DacA_CD_ using a previously reported coupled enzyme assay [40], no significant enzyme inhibition was observed. The specific activity of the pure GlmM enzyme was estimated at 15.9 ± 3.1 μmol/min/mg of protein in the assay conditions used. Addition of DacA in a 5- to 50-fold excess over GlmM did not at all inhibit the phosphoglucosamine mutase activity and its presence in 400-fold excess only led to a slight 15% reduction of GlmM activity. Taken together, these data indicate that only GlmM can impact the activity of the c-di-AMP cyclase enzyme but likely not *vice versa*.

### GlmM of Gram-negative bacteria do not regulate or interact with the *S*. *aureus* DacA protein

**Fig 6:**
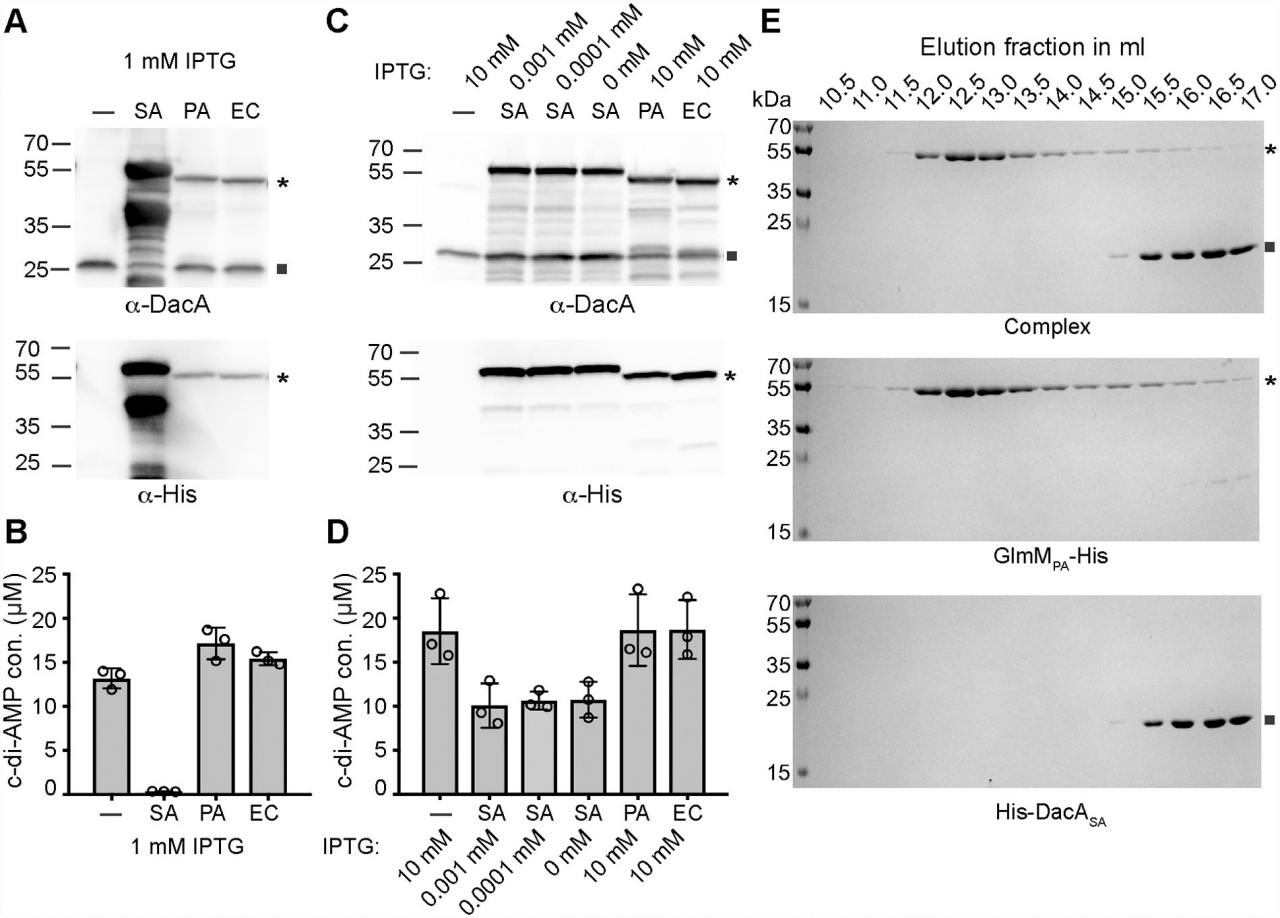
GlmM proteins from *E*. *coli* or *P*. *aeruginosa* do not interact or impact the activity of the *S*. *aureus* DacA enzyme. (A and C) Detection of DacA and GlmM-His by western blot. Protein samples were prepared from *E*. *coli* XL1-Blue pBAD33-*dacA* containing either an empty p*Trc*His60 vector (-) or a plasmid for expression of the *S*. *aureus* GlmM-His (SA), the *P*. *aeruginosa* GlmM-His (PA) or the *E*. *coli* GlmM-His (EC). DacA or the GlmM-His proteins were detected by western-blot using an anti-DacA or anti-His antibody as indicated below the respective panel. DacA protein expression was induced with 0.2% arabinose and GlmM protein expression with 1 mM IPTG in (A) or with the amount of IPTG as indicated in (C). (B and D). ELISA determination of c-di-AMP levels in *E*. *coli*. Cell extracts were prepared from the *E*. *coli* strains used in panels A and C. The cellular c-di-AMP levels in μM per ml *E*. *coli* culture with an A_600_ of 10 were determined by ELISA and the average values and standard deviations of three independent experiments plotted. (E) Coomassie stained gels. Aliquots from the size exclusion fractions of the complex (top), the *P*. *aeruginosa* GlmM_PA_-His (middle) or the *S*. *aureus* His-DacA_SA_ (bottom) were separated on 12% PAA gels and proteins visualized by Coomassie staining. The experiment was performed twice, and a representative result is shown. GlmM and DacA proteins are indicated with an asterisk and a square, respectively in panels A, C and E.

Next, we tested if GlmM proteins from unrelated Gram-negative bacteria can also interact and influence the activity of the *S*. *aureus* DacA protein. To this end, we overexpressed the *S*. *aureus* DacA protein from pBAD33-*dacA* along with C-terminally His-tagged versions of the *E*. *coli* (EC), *Pseudomonas aeruginosa* (PA) and as control *S*. *aureus* (SA) GlmM proteins in *E*. *coli* and measured c-di-AMP levels by ELISA (Fig 6). As expected, expression of the *S*. *aureus* GlmM-His protein (SA) blocked c-di-AMP production, while c-di-AMP levels were not reduced upon expression of the *E*. *coli* or *P*. *aeruginosa* GlmM proteins (Fig 6B). However, when we analyzed the GlmM protein levels by western-blot following induction with 1 mM IPTG, we noted that the *S*. *aureus* proteins was expressed at significant higher levels as compared to the other two GlmM proteins (Fig 6A). Therefore, we repeated the experiment using 10 mM IPTG for the induction of the *E*. *coli* and *P*. *aeruginosa* GlmM proteins but no (0 mM) or very low levels (0.001 or 0.0001 mM) of IPTG for the induction of the *S*. *aureus* GlmM protein. In this case, similar GlmM amounts were observed for all strains (Fig 6C). Again, only expression of the *S*. *aureus* GlmM protein (be it to a lesser extent) but not expression of the GlmM proteins from the Gram-negative bacteria led to a reduction in the cellular c-di-AMP levels (Fig 6D). Next, we investigated the interaction of the *S*. *aureus* DacA and *P*. *aeruginosa* GlmM proteins *in vitro*. The expression of the *S*. *aureus* His-DacA_CD_ and *P*. *aeruginosa* GlmM-His proteins were induced in *E*. *coli*, the cells lysed and the individual proteins purified by Ni-affinity followed by size exclusion chromatography. In addition, the lysates from the *S*. *aureus* His-DacA_CD_ and *P*. *aeruginosa* GlmM-His overexpressing strains were mixed before affinity and size exclusion chromatography. In contrast to the observation with the *S*. *aureus* GlmM protein (Fig 4A), the *P*. *aeruginosa* GlmM-His protein did not coelute with the *S*. *aureus* His-DacA_CD_ (Fig 6E). Taken together, these data highlight a specificity in the interaction between the *S*. *aureus* DacA and GlmM proteins, as no interaction was detected between the *S*. *aureus* DacA protein and the GlmM proteins from the Gram-negative bacteria *E*. *coli* and *P*. *aeruginosa*.

### *S*. *aureus* GlmM protein structure and small-angle X-ray scattering analysis of the DacA_CD_/GlmM complex

**Fig 7:**
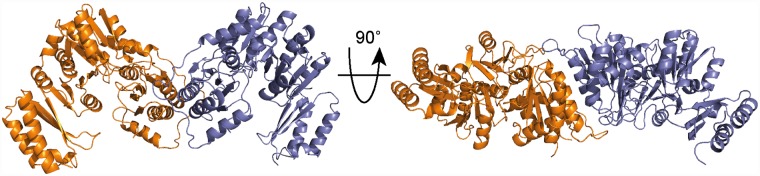
*S*. *aureus* GlmM structure. Structure of the *S*. *aureus* GlmM protein shown in ribbon representation. The protein was crystallized in the presence of GlcN-6P and MgCl_2_ but no densities for the metal ion or substrate were present in the structure. The structure was solved by molecular replacement using the *B*. *anthracis* GlmM protein (PDB 3PDK) as template. The protein crystallized as dimer and the two different monomers are shown in orange and purple. The dimer is shown in two views with the second view rotated by 90°.

**Fig 8:**
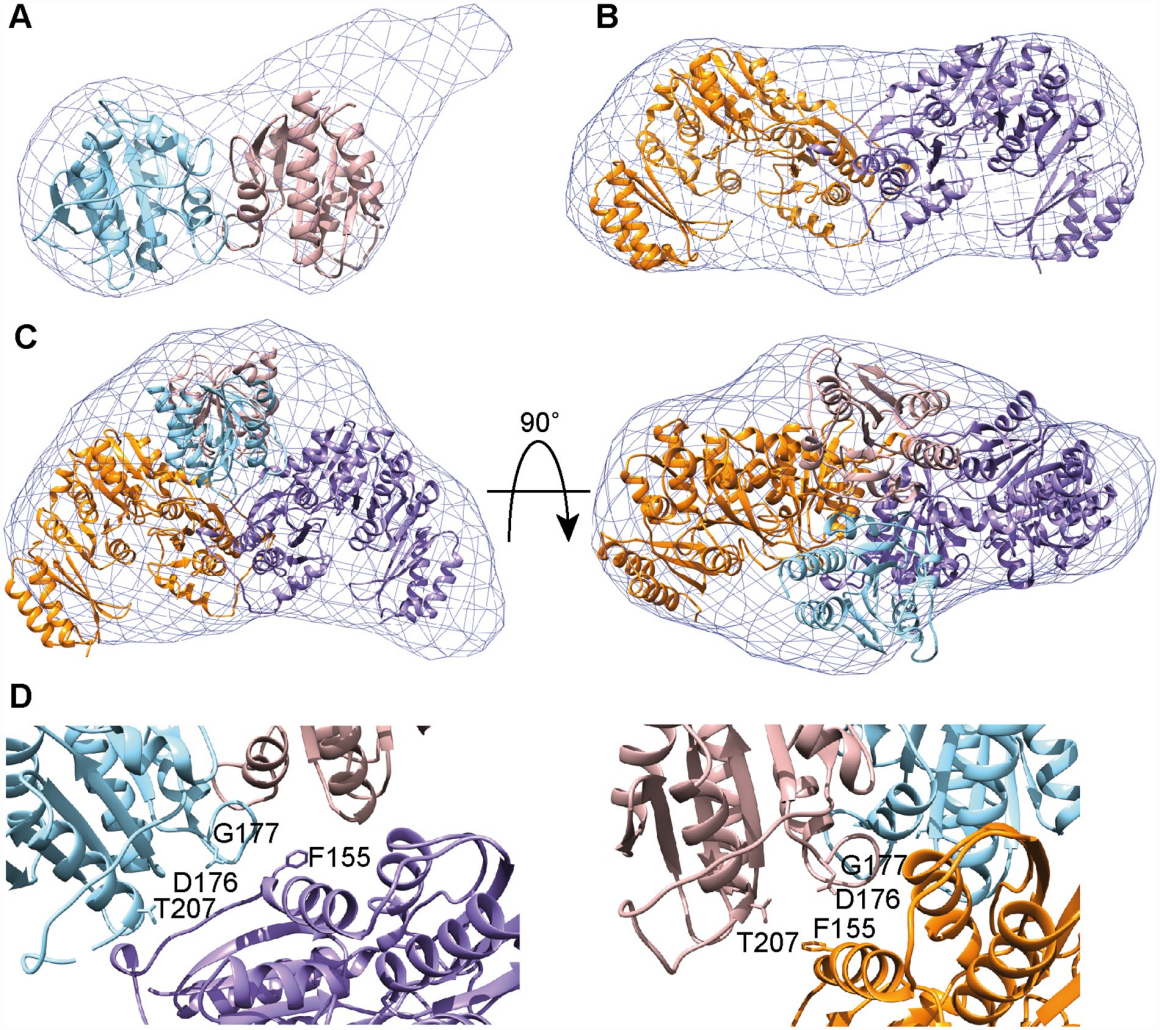
Small Angle X-ray Scattering (SAXS) data for *S*. *aureus* proteins DacA_CD_, GlmM and the DacA_CD_/GlmM complex. Reconstructed envelopes of the purified DacA_CD_ (A), GlmM (B) and DacA_CD_/GlmM complex (C). DacA_CD_ (12 mg/ml), GlmM (8 mg/ml) and the DacA_CD_/GlmM complex (12 mg/ml) were injected onto a Superdex 200 5/150 column coupled to a Small-Angle X-Ray beam. All data were analyzed and envelopes reconstructed using ScÅtter. Conversion of envelopes to maps and subsequent structure fitting were performed using Chimera. (D) Zoomed in views of the DacA_CD_ active sites in the DacA_CD_/GlmM SAXS model. Catalytic residues on both DacA_CD_ protomers are shown in stick representations. The interaction of GlmM with DacA_CD_ covers the active sites, thus preventing DacA_CD_ dimers from interacting and forming catalytically active species.

In order to gain structural information on the *S*. *aureus* DacA_CD_/GlmM complex, we first set out to determine the structure of the *S*. *aureus* GlmM protein in isolation. To this end, the C-terminal His-tagged *S*. *aureus* GlmM protein was purified, the His-tag was removed, and the purified protein crystallized in the presence of Mg^2+^ and glucosamine-6-P. The structure of GlmM was obtained by molecular replacement using the GlmM protein structure from *Bacillus anthracis* (PDB 3PDK) as a search model [41] (Fig 7 and S1 Table). While the protein was crystallized in the presence of metal ion and substrate, no corresponding extra electron density was observed. The *S*. *aureus* GlmM protein and the *B*. *anthracis* protein, which share 67% identity on a protein level, displayed a similar fold and dimeric arrangement and the structures overlapped with an RMSD of 0.996 Å. One GlmM monomer consisted of four α-β domains with the fourth most C-terminal domain linked by a flexible loop (Fig 7). Next, we attempted to crystallize and solve the structure of the *S*. *aureus* DacA_CD_/GlmM complex. While crystals were obtained under several conditions, poor diffraction prevented the structural determination of the complex. In order to gain structural information of the DacA/GlmM complex, the single proteins and complex were subjected to small-angle X-ray scattering (SAXS) analysis (Fig 8, S6 and S7 Figs and S3 Table). The reconstructed model for the GlmM sample showed an elongated particle, consistent with the dimeric arrangement found in the crystal structure (Fig 8B) and with previously published findings [42]. In the case of DacA, the model was most consistent with the particle being a DacA_CD_ dimer, however an extra density was observed to one extremity (Fig 8A), which we speculate is due to the presence of extra N-terminal amino acids, not visible in the X-ray structure, and hence appear as a flexible and unstructured region of the protein. The reconstructed envelope of the complex displays a bigger particle size as compared to GlmM dimer alone, consistent with a DacA_CD_ dimer interacting with a GlmM dimer (Fig 8C). A sequential fitting of the GlmM dimer structure followed by the fitting of the DacA dimer structure into the SAXS envelope of the complex with Chimera [43] yielded the best fit for a model with a correlation score of 0.9196 in which a DacA dimer is positioned on top of a GlmM dimer (Fig 8C). In this model, the active sites of DacA protomers are occluded through binding to the GlmM protein (Fig 8D), while the GlmM active sites are still accessible. To validate the SAXS analysis, the reconstructed envelopes were used to calculate theoretical Collision Cross Section (CCS) values using the program EM∩IM [44]. These theoretical values were subsequently compared to those experimentally determined by Ion-Mobility Mass Spectrometry. The theoretical values agree with the CCS determination (S4 Table), showing that the structural models of DacA, GlmM and their complex obtained by SAXS correspond to the shapes of the proteins in solution. Taken together, our model provides an explanation as to how the interaction of DacA with GlmM inhibits c-di-AMP production without affecting the activity of the GlmM enzyme.

## Discussion

In this study, we have determined the structures of the *S*. *aureus* enzymes DacA and GlmM and provide structural and functional information on the complex formed between these two enzymes. Previously, these enzymes have been shown to interact, and using the information obtained here, we can now propose a speculative molecular mechanism for the regulation of the cyclase activity of DacA by GlmM. Specifically, our data suggest that GlmM blocks access to the active site of DacA and in this manner prevents the formation of catalytically active head-to-head DacA oligomers.

c-di-AMP production is essential for the growth of *S*. *aureus* under standard laboratory conditions [20, 45] and the only enzyme in this bacterium that produces c-di-AMP is DacA. The *S*. *aureus* DacA_CD_ catalytic domain investigated as part of this study has, as expected, a very similar overall fold to the enzymatic domain of the *L*. *monocytogenes* CdaA cyclase [35]. However, there were also notable and interesting differences. As expected, the side chains of amino acids D176 and G177 in the HDG motif and T207 before the RHR motif in the *S*. *aureus* DacA_CD_ enzyme made contacts with the ribose and the phosphate backbone of ApCpp, respectively (Fig 1). However, in addition to these residues, we also observe an additional π-stacking interaction between residue Y192 and the adenine base (Fig 1) in the DacA_CD_-ApCpp structure. This residue is located in a loop region between ß4 and α4. A structure-based alignment using STRAP [46] showed that this residue is not conserved in *T*. *maritima* and *B*. *subtilis* DisA proteins, or in *B*. *cereus* CdaS, but present in *L*. *monocytogenes* CdaA (S8 Fig). This indicates that this residue could potentially play an important role in fine-tuning c-di-AMP production in *S*. *aureus* and other DacA/CdaA-containing bacteria by affecting the binding of the nucleotide substrate.

**Fig 9:**
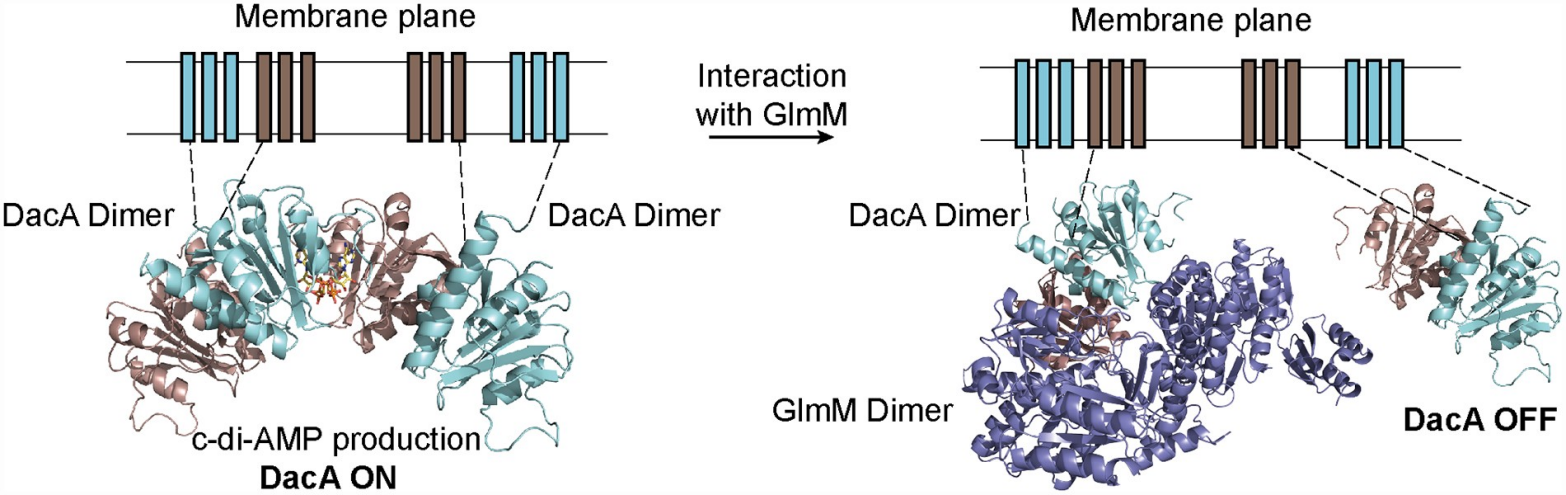
Proposed model of DacA inhibition by GlmM. (Left) For active c-di-AMP synthesis, two (or more) DacA dimers interact forming catalytically active head-to-head oligomers. (Right) When GlmM binds to a DacA dimer, we speculate that this will prevent DacA from forming higher oligomers thereby turning off c-di-AMP production. GlmM dimer is colored in purple and a DacA dimer is colored in cyan (chain A) and brown (chain B). Bound ATP is colored in yellow. DacA transmembrane helices are modeled on the membrane plane and connected to the N-terminal helices of the DacA_CD_ domain by dashed lines.

Similar to other c-di-AMP cyclases, which require a metal ion as cofactor (usually Mg^2+^, Mn^2+^ and/or Co^2+^) [23, 35, 47], we found that *S*. *aureus* DacA_CD_ has the highest *in vitro* enzyme activity in the presence of Mn^2+^, followed by Mg^2+^ and was least active in the presence of Co^2+^ (Fig 3). This is very similar to what has been described for the *Mycobacterium tuberculosis* c-di-AMP cyclase Rv3586 (a DisA family enzyme), but differs from what has been reported for the *L*. *monocytogenes* CdaA enzyme, which was most active in the presence of Co^2+^, whereas Mg^2+^ did not support enzyme catalysis [35, 47]. In addition to ATP, for some c-di-AMP cyclases it has been shown that ADP can also be used as substrate [47]. Interestingly, when the *M*. *tuberculosis* c-di-AMP cyclase Rv3586 was provided with ADP as substrate, the highest activity was seen in the presence of Mg^2+^, rather than Mn^2+^ [47]. While the actual metal preference and requirement for the activity of c-di-AMP cyclases *in vivo* is not known, it is conceivable that metal ion availability as well as substrate availability (ATP versus ADP) might be an important factor in adjusting c-di-AMP levels in the cell.

c-di-AMP cyclase enzymes form higher-order oligomeric complexes in solution. The CdaS- and DisA-type enzymes form hexamers and octamers, respectively [23, 33, 34], whereas the catalytic domain of the *L*. *monocytogenes* cyclase CdaA was reported to be a dimer in solution [35]. Indeed, for the c-di-AMP condensation reaction to take place, the enzyme requires two catalytic sites in a symmetric head-to-head conformation. Here, we show that the *S*. *aureus* DacA_CD_ protein is a dimer (S2 Fig and Fig 8). However, based on the data presented in this study, we suggest that this dimer is not the active unit per se, as the protomers are not found in a head-to-head conformation (Fig 1). We propose that multiple dimers need to form higher oligomers (e.g. tetramers) in order to produce c-di-AMP (Figs 3 and 9). Therefore, by regulating the ability of DacA_CD_ to form higher oligomers its activity can be regulated and as discussed below, we proposed that this is the mechanism by which GlmM inhibits the activity of DacA.

The same inactive (non-head-to-head) dimer conformation as observed in our DacA_CD_ structure has also been observed in the hexamer model of the CdaS di-adenylate cyclase from *Bacillus* spp. (PDB 2FB5) [33] (S9 Fig). Mehne *et al*. proposed that the hexamerization of CdaS is driven by two sets of interactions: the interactions between the two N-terminal helices (YojJ domain) and the interactions between residues found at the dimer interface between two DAC domains. The latter interactions are similar the ones we observed in the *S*. *aureus* DacA_CD_ dimer (Fig 1 and S9 Fig) [33]. After deletion of the N-terminal helices, the *B*. *subtilis* CdaS enzyme formed dimers in solution and this truncated protein had high catalytic activity [33]. However, it was not further investigated if the CdaS dimers were the active unit or if they still needed to undergo higher oligomerization to be catalytically active, similar to what we observed for the *S*. *aureus* DacA_CD_ enzyme. While for the DisA-type enzyme it seems clear that the enzymatically active form is an octamer [23], additional studies are needed to determine in which oligomeric state CdaS- and CdaA/DacA-type c-di-AMP cyclases function *in vivo*.

The membrane-anchored DacA/CdaA-type c-di-AMP cyclase enzyme is encoded in a conserved three or four gene operon together with the predicted cyclase regulator YbbR (CdaR) and the phosphoglucosamine mutase enzyme GlmM producing the essential peptidoglycan synthesis intermediate glucosamine-1-P [24, 48]. Indeed, for *S*. *aureus* it has been shown that all three genes are co-expressed from an upstream promoter [49]. However, a second internal promoter is present in front of *glmM* suggesting that *S*. *aureus* produces higher GlmM protein levels as compared to DacA and YbbR [49]. It is thought that both GlmM and YbbR can regulate the activity of the cyclase DacA/CdaA [17, 18]; however, for *S*. *aureus* we could not find conclusive evidence for a regulatory function of YbbR in this nor in a previous study (Fig 5) [12]. Recently, the first experimental evidence that the c-di-AMP cyclase DacA (CdaA), GlmM and YbbR (CdaR) form a three-protein complex was obtained in *B*. *subtilis* and *L*. *lactis* [17, 18]. The data presented here provide the first structural insight into this regulatory complex and further mechanistic information. Consistent with the *in vivo* work in *L*. *lactis* [17], we show that the *S*. *aureus* GlmM protein (but not the *E*. *coli or P*. *aeruginosa* GlmM proteins) is a strong negative regulator of the *S*. *aureus* cyclase DacA_CD_ (Fig 5). Since we performed this work *in vitro* with purified proteins, it shows that no additional components are required for this inhibitory function.

Our SAXS envelope data are most consistent with the formation of a complex in which a DacA_CD_ dimer sits on top of the GlmM dimer, forming a dimer of dimers (Fig 8). The resolution of our SAXS reconstruction is too low to infer specific amino acid interactions between DacA_CD_ and GlmM or to assess any conformational changes of DacA_CD_ upon GlmM binding. However, the data are of sufficient quality to construct models of the of DacA_CD_-GlmM complex. In our model, the DacA_CD_ active sites in both protomers are capped by the α_5_ helix of GlmM and neighbouring residues (Fig 8D). Hence the interaction of the GlmM dimer with the DacA_CD_ dimer will prevent DacA_CD_ from forming higher oligomers required for activity. In addition, the interaction of GlmM with DacA_CD_ could potentially block the binding of substrate to DacA_CD_. Interestingly, positioned next to the α_5_ helix in GlmM is residue F155, which in a previous study was shown to modulate the GlmM-CdaA interaction in *L*. *lactis* [17]. Our SAXS-based model provides now a molecular rational as to why a residue in this position could modulate complex formation and hence cyclase activity.

Within bacterial cells, the GlmM/DacA complex will likely be dynamic and in this manner allowing for c-di-AMP levels to be adjusted based on to environmental signals. It is conceivable that depending on the cellular levels of the substrate or product for GlmM (glucosamine-6-P and glucosamine-1-P, respectively), the ability of GlmM to interact with DacA changes and that in this manner c-di-AMP production is synchronized with the production of peptidoglycan precursors. GlmM functions in a pathway together with GlmS, which produces the GlmM substrate glucosamine-6-P and converts as part of this reaction glutamine to glutamate, and GlmU, which acts after GlmM and produces UDP-*N*-acetylglucosamine. It is also possible that the GlmM/DacA interaction could be affected by the activity of GlmS and GlmU and availability of their substrates. This possibility is intriguing, as previous studies have revealed an impact of cellular glutamine, glutamate and UDP-*N*-acetylglucosamine levels on the cellular c-di-AMP levels [17, 18, 50].

Taken together, our results provide the first structural characterization of the DacA/GlmM complex providing mechanistic insight into how c-di-AMP production is controlled directly at the synthesis level through the binding of GlmM to DacA. Additional work is needed to better understand the dynamics of this complex, in particular in the context of the bacterial membrane and how this regulatory complex can be modulated by cellular or external stimuli.

## Materials and methods

### Bacterial strains and plasmid construction

All strains used in this study are listed in S5 Table and primers used for plasmid construction are listed in S6 Table. *E*. *coli* strains were grown in Lysogenic broth (LB) supplemented when appropriate with the antibiotics indicated in S5 Table. Protein expression was induced with 0.5 mM IPTG unless specified otherwise in the text or 0.2% arabinose. Plasmids for the expression of the *S*. *aureus* DacA and GlmM proteins were constructed as follows: The DNA fragments coding for the *S*. *aureus* DacA catalytic domain starting from amino acid residue F101 (referred to as DacA_CD_ domain) and GlmM were amplified by a PCR using LAC* genomic DNA as template and primer pairs ANG1135/ANG1137 (for DacA_CD_) or ANG2342/ANG2343 (for GlmM). The primers for cloning *glmM* contained DNA sequences for appending a C-terminal 6-His tag proceeded by a thrombin cleavage site. The PCR products were digested with NheI/EcoRI (for DacA_CD_) or NcoI/XhoI (for GlmM-His) and then ligated with plasmid pET28b, which has been digested with the same enzymes. The resulting plasmids pET28b-His-*dacA*_*CD*_ and pET28b-*glmM*-His were initially recovered in *E*. *coli* XL1-Blue yielding strains ANG1858 and ANG3994 and subsequently introduced into *E*. *coli* BL21 (DE3) yielding the protein overexpression strains ANG1865 and ANG3997, respectively. Plasmids pET28b-His-*dacA*_*CD*_-K and pET28b-His-*dacA*_*CD*_-C for the expression of the DacA_CD_ variants with amino acid residues N166 and T172 replaced by lysines (DacA_CD_-K variant) or cysteines (DacA_CD_-C variant) were constructed by Splicing Overhang Extensions (SOE) PCR. Primer pairs ANG1135/ANG2616 and ANG2617/ANG1137 (DacA_CD_-K variant) or ANG1135/ANG2618 and ANG2619/ANG1137 (DacA_CD_-C variant) were used for the first PCR. Next, the respective PCR products were fused in a second PCR using primer ANG1135 and ANG1137. The resulting products were digested with NheI/EcoRI and ligated with pET28b that had been cut with the same enzymes. The resulting plasmids pET28b-His-*dacA*_*CD*_-K and pET28b-His-*dacA*_*CD*_-C were initially recovered in *E*. *coli* XL1-Blue giving strains ANG4319 and ANG4321 and subsequently introduced in *E*. *coli* BL21 (DE3) yielding strains ANG4320 and ANG4322. For construction of plasmids pBAD33-*dacA*, pBAD33-*dacA-ybbR* and pBAD33-*dacA-ybbR-glmM* the *dacA*, *dacA-ybbR* and *dacA-ybbR-glmM* regions were amplified by PCR from *S*. *aureus* LAC* chromosomal DNA using primer pairs ANG928/ANG2475, ANG928/ANG2476 and ANG928/ANG2477, respectively. The PCR products were cut with KpnI and HindIII and ligated with plasmid pBAD33, which had been cut with the same enzymes. The resulting plasmids pBAD33-*dacA*, pBAD33-*dacA-ybbR* and pBAD33-*dacA-ybbR-glmM* were recovered in *E*. *coli* XL1-Blue yielding strains ANG4120, ANG4121 and ANG4122. For construction of plasmid pBAD33-*dacA-no-ybbR-glmM* (strain ANG4263), the YbbR start codon was mutated by SOE PCR. To this end, the front and back parts of the *dacA-no-ybbR-glmM* insert were amplified using plasmid pBAD33-*dacA-ybbR-glmM* as template and primer pairs ANG1244/ANG2597 and ANG2598/ANG1245, respectively. The PCR fragments were fusing in a second PCR using primer pair ANG1244/ANG1245. The resulting product was digested with KpnI and HindIII and cloned into plasmid pBAD33 cut with the same enzymes. The sequences of inserts were verified by fluorescent automated sequencing at GATC Biotech. For the overexpression of the *S*. *aureus* DacA enzyme and His-tagged GlmM proteins from different bacteria, the empty control plasmid p*Trc*His60, as well as plasmids pMLJ4 (GlmM-His *E*. *coli*), pMLJ11 (GlmM-His *S*. *aureus*) or pMLD137 (GlmM-His *P*. *aeruginosa*) were introduced into *E*. *coli* strain XL1-Blue pBAD33-*dacA*, yielding strains ANG5174, ANG5175, ANG5177 and ANG5178, respectively.

### Protein expression, purification, and histidine-tag cleavage

One to two litre cultures of *E*. *coli* strains BL21 (DE3) containing plasmids pET28b-His-*dacA*_*CD*_ (ANG1865), pET28b-His-*dacA*_*CD*_-K (ANG4320), pET28b-His-*dacA*_*CD*_-C (ANG4322) or pET28b-*glmM*-His (ANG3997) were grown in LB medium supplemented with kanamycin (30 μg/ml) at 37°C with agitation. Once the cultures reached an A_600_ of approximately 0.5–0.6, protein expression was induced with 0.5 mM IPTG and the cultures were subsequently incubated for a further 3 hours at 37°C. Cells were harvested by centrifugation, suspended in 20 ml of 50 mM Tris pH 7.5, 500 mM NaCl buffer and lysed by passing the cell suspensions twice through a French press cell at 1100 psi. For the purification of the His-DacA_CD_/GlmM-His complex, the appropriate cell lysates were mixed after lysis and incubated on ice for 10 minutes. Next, the lysates were clarified by centrifugation at 20,000 × *g* for 40 min and then loaded by gravity flow onto columns containing 4 ml Ni-NTA resin (Qiagen) equilibrated prior to use with 50 mM Tris pH 7.5, 500 mM NaCl buffer. The columns were washed with 30 ml of 50 mM Tris pH 7.5, 500 mM NaCl, then 30 ml of 50 mM Tris pH 7.5, 500 mM NaCl, 50 mM imidazole buffer and the proteins eluted in 5 × 1 ml fractions with 50 mM Tris pH 7.5, 200 mM NaCl, 500 mM imidazole buffer. Elution fractions containing the purified protein or protein complex were pooled and then loaded onto a Superdex 200 10/60 Hiload column (GE Healthcare), equilibrated with 30 mM Tris pH 7.5, 150 mM NaCl buffer. When the GlmM-His protein was purified alone, DTT was added to the purified protein at a final concentration of 1 mM after the size-exclusion chromatography step to maintain the protein in a soluble state. To cleave the His-tag from proteins, 20 U thrombin (Sigma) was added per 10 mg protein after the Ni-NTA column purification step and the mixture was incubated overnight at 4°C with agitation. The tag-less proteins were subsequently purified by size exclusion chromatography as described above. The purified proteins or complex were concentrated using 10 kDa cutoff Amicon centricons (Millipore). Protein concentration was measured either by determining the absorbance at 280 nm or by the Bradford method. For the investigation of the interaction between the *S*. *aureus* His-DacA_CD_ enzyme and the *P*. *aurugniosa* GlmM-His, one litre cultures of *E*. *coli* strain BL21 (DE3) containing plasmid pET28b-His-*dacA*_*CD*_ (ANG1865) was grown in LB medium supplemented with kanamycin (30 μg/ml) at 37°C with agitation. Once the culture reached an A_600_ of 0.5, protein expression was induced with 0.5 mM IPTG and the culture was subsequently incubated O/N at 16°C. Strain XL1-Blue pMLD137 (GlmM-His *P*. *aeruginosa*) was grown to an A_600_ of 0.5 and protein expression was induced with 0.5 mM IPTG and the culture subsequently incubated for an additional 4 hours at 37°C. Cells were harvested by centrifugation, suspended in 20 ml of 50 mM Tris pH 7.5, 500 mM NaCl buffer supplemented with 0.1% v/v ß-mercaptoethanol and complete EDTA free protease inhibitor (Roche) and lysed by sonication for 2 min at 15 sec on and 30 sec off intervals at 60% amplitude. The proteins and complex were purified by nickel affinity chromatography as described above but using only 2 ml of Ni-NTA resin and eluting the proteins in 2.5 ml of 50 mM Tris pH 7.5, 200 mM NaCl, 500 mM imidazole buffer supplemented with 0.1% v/v ß-mercaptoethanol. Next, 500 μl of the eluate was separate on a Superdex 200 10/300GL column (GE Healthcare) using 30 mM Tris pH 7.5, 150 mM NaCl as running buffer. 0.5 ml fractions were collected, and aliquots of the indicated elution fractions separated on 12% PAA gels and proteins visualized by Coomassie staining.

### SEC-MALS and native mass spectrometry analysis of the DacA_CD_/GlmM protein complex

100 μl of the purified, tag-less DacA_CD_/GlmM protein complex at 18 mg/ml was loaded onto a Superdex 200 Increase 10/300 column (GE Healthcare) pre-equilibrated with 30 mM Tris pH 7.5, 150 mM NaCl buffer and run at 0.5 ml/min in the same buffer. The column was coupled to a MALS detector and refractometer and the UV absorbance, laser scattering and refractive index change were monitored throughout the run. The data were analysed using the ASTRA 6.0 software and fitted according to the Zimm model for static light scattering. The experiment was performed in duplicate and a representative graph is shown. Prior to the native mass spectrometry experiment, the tag-less DacA_CD_/GlmM was loaded onto a Superdex200 10/300 column (GE Healthcare) pre-equilibrated with 200 mM ammonium acetate pH 7.5. Samples were further buffer exchanged with Amicon Ultra centrifugal filtration units (Merck Millipore) using 200 mM Ammonium Acetate (Fisher Scientific). Samples were diluted to 13.5 μM. The samples were directly introduced into a first generation Waters Synapt QToF (Waters Corporation, UK) using nanoelectrospray gold-coated borosilicate glass capillaries prepared *in-house* [51]. Mass calibration was performed using 30 mg/mL Caesium Iodide (Fluka) solution. Machine parameters used were: capillary 1.4 kV, sampling cone 40 V, extraction cone 4 V, backing pressure 6.20 mbar, trap CE 15 eV, transfer CE 13 eV, bias 16 V, source wave velocity 300 ms^-1^, source wave height 0.2 V, trap wave velocity 300 ms^-1^, trap wave height 0.2 V, IMS wave velocity 260 ms^-1^, IMS wave height 8.0 V, transfer wave velocity 260 ms^-1^, transfer wave height 8.0 V. For collision cross section calculation calibrants were sprayed from 200mM Ammonium Acetate solution. The calibrants used were Cytochrome C (Calbiochem), β-Lactoglobulin (Sigma Aldrich), Bovine Serum Albumin (Sigma Aldrich), Concanavalin A (Sigma Aldrich), Serum P Albumin (Calbiochem) and Pyruvate Kinase (Sigma Aldrich). The CCS calibration curve was produced using the method described by Thalassinos *et al*. [52], with a calculated R^2^ value of 0.9601. Spectra were analyzed using MassLynx v4.1 (Waters Corporation, UK) and the program Amphitrite [53].

### Protein crystallization, structure solution and analysis

The tag-less, nucleotide-free DacA_CD_ protein and tag-less DacA_CD_-C variant were crystallized in 200 mM LiCl_2_, 100 mM Na-cacodylate pH 6.5, 30% PEG400, using a protein concentration of 7.5 mg/ml. To obtain the ApCpp-bound DacA_CD_ complex structure, the tag-less DacA_CD_ protein at a concentration of 7.5 mg/ml was incubated for 30 min on ice with 2 mM ApCpp (Jena Bioscience) and 2 mM MnCl_2_ and the protein subsequently crystallized in 10 mM MgCl_2_, 50 mM MES pH 5.8, 0.2M KCl and 3% PEG8000 buffer. The tag-less GlmM protein was crystallized in the presence of 2 mM MgCl_2_ and 2 mM GlcN-6P (Sigma) in 2.0 M sodium malonate buffer. All proteins were crystallized by the vapour diffusion method. DacA_CD_, DacA_CD_-C and GlmM crystals were frozen in liquid nitrogen without the addition of further cryo-protectants and datasets collected at the I03 Beamline at the Diamond Light Source (Harwell Campus, Didcot, UK). Data integration was performed with DIALS [54], the reflections were scaled and merged with Aimless [55] and intensities were converted to structure factors using Ctruncate [56]. Analysis of the Matthews coefficient revealed that two protein molecules were present in the asymmetric unit for all proteins. The structures were solved by the molecular replacement method with Phaser [57] using the *L*. *monocytogenes* CdaA protein (PDB 4RV7) as a template for DacA_CD_ or the *B*. *anthracis* GlmM protein (PDB 3PDK) as a template for the *S*. *aureus* GlmM protein. Structure refinement was performed with Phenix [58] and model building with Coot [59]. Structure figures were created with Pymol and Chimera [43, 60].

### Affinity pull-down experiments

For the pull-down experiment, 50 μM tag-less DacA_CD_ and 50 μM GlmM-His protein were mixed in 30 mM Tris pH 7.5, 150 mM NaCl buffer and incubated on ice for 10 min. Next, the protein mixture was applied by gravity flow to a column containing 1 ml of Ni-NTA equilibrated with 30 mM Tris pH 7.5, 150 mM NaCl buffer. As a control, 500 μl of a 50 μM tag-less DacA protein solution in 30 mM Tris pH 7.5, 150 mM NaCl were also applied by gravity flow onto a column. The flow-through fractions were collected for subsequent SDS-PAGE analysis. The columns were then washed with 15 ml of 30 mM Tris pH 7.5, 150 mM NaCl, 10 mM imidazole buffer and the wash fraction collected and proteins finally eluted with 2 ml 30 mM Tris pH 7.5, 150 mM NaCl, 500 mM imidazole buffer. Aliquots of the load, flow through, wash and elution fractions were run on a 12% SDS-PAGE gel and proteins visualized by Coomassie staining. The DacA_CD_/GlmM-His pulldown experiment was performed in triplicate and a representative result is shown; the control experiment using the DacA_CD_ protein alone was performed once.

### DacA_CD_ enzyme activity assay

To assess the metal dependency of the *S*. *aureus* DacA_CD_ enzyme, enzyme reactions were set up as follows: 2 μl of 10X reaction buffer (400 mM Hepes pH 7, 1 M NaCl, 100 mM or 10 mM of MgCl_2_, CoCl_2_ or MnCl_2_) was mixed on ice with 13.6 μl of water and 2 μl of 1 mM ATP. Next, 2 μl of DacA_CD_ from a 50 μM stock solution and 0.4 μl of α-P^32^-labelled ATP (Perkin Elmer—3.3 μM, 250 μCi) were then added to the sample yielding a final reaction volume of 20 μl. The reactions were incubated at 37°C for 4 h. Reactions were stopped by heating for 5 min at 95°C and the conversion of ATP to c-di-AMP assessed by spotting 1 μl of each reaction onto a TLC plate (Millipore). The nucleotides were separated using a 3.52 M (NH_4_)_2_SO_4_ and 1.5 M KH_2_PO_4_ (mixed in a 1:1.5 v/v ratio) buffer system, the TLC plate were subsequently dried and the radioactive signal detected using a Typhoon FLA 700 imager. The bands corresponding to radioactive ATP and c-di-AMP were quantified using the ImageQuant software and the % ATP to c-di-AMP conversion calculated. As the highest enzyme activity was seen in the presence of MnCl_2_, all subsequent enzyme reactions were set up in reaction buffer containing a final concentration of 10 mM MnCl_2_. For the time course experiment, 100-μl reactions were set and 10-μl aliquots removed at the indicated time point and stopped by heating. The activity of the DacA_CD_-K and DacA_CD_-C variants was assessed by setting up 20-μl reactions as described above using a final protein concentration of 5 μM and incubating the reactions for 4 h at 37°C. To analyze the ATP conversion of DacA_CD_ when bound to GlmM, 2 μl of GlmM from a 100 μM stock solution was added to a standard enzyme reaction yielding a DacA_CD_:GlmM molar ratio of 1:2 and the reactions were incubated for 4 h at 37°C. To evaluate the impact of the addition of GlmM when added to an ongoing DacA_CD_ enzyme reaction, 2 μl of GlmM from a 100 μM stock solution were added 30 min after initiating the reaction and the reaction was incubation for a further 2 h. As control, DacA_CD_ reactions set up in the absence of GlmM were stopped after 30 min and 3 h. The enzyme assays were performed in triplicate and the average values and standard deviations plotted.

### GlmM enzyme activity assay

The phosphoglucosamine mutase activity of GlmM was determined by using a coupled radioactive assay in which the GlcN-1-P synthesized from GlcN-6-P by GlmM was quantitatively converted to *N*-acetylglucosamine-1- phosphate (GlcNAc-1-P) in the presence of pure GlmU enzyme [40]. The standard assay mixture (50 μl) contained 50 mM Tris/HCl (pH 8), 3 mM MgCl_2_, 1 mM GlcN-6-P, 0.4 mM [^14^C]acetyl-CoA (1.9 GBq/mmol, 700 Bq), 0.7 mM glucose-1,6-diphosphate, pure *E*. *coli* GlmU (30 ng) and *S*. *aureus* GlmM (10–50 ng) enzymes (appropriate dilutions in 20 mM phosphate buffer, pH 7.2, containing 0.5 mM MgCl_2_ and 0.1% 2-mercaptoethanol). When the DacA protein was also included in the assay, pure GlmM and DacA proteins were first pre-incubated together with different ratios at 37°C for 5 min before addition to the reaction mixture at t = 0. Then, mixtures were incubated at 37°C for 30 min and the reactions were stopped by the addition of 10 μl of glacial acetic acid. The radioactive substrate (acetyl-CoA) and product (GlcNAc-1-P) were separated by thin-layer chromatography (TLC) on pre-coated silica gel 60F_254_ plates (Merck) using 1-propanol/ammonium hydroxide/water (6:3:1, v/v) as the mobile phase (*R*_*f*_ factors of these compounds were 0.72 and 0.17, respectively). Radioactive spots were located and quantitated with a radioactivity scanner (Rita Star, Raytest Isotopenmeβgeräte GmbH, Straubenhardt, Germany).

### Assessment of protein stability by thermofluor analysis

To assess the protein stability, a thermofluor experiment was performed. Reactions were set up as follows: 2 μl of either 10X enzyme reaction buffer (400 mM Hepes pH 7, 1 M NaCl, 100 mM MnCl_2_) or 10X protein purification buffer (300 mM Tris pH 7.5, 1.5M NaCl) were added to the appropriate wells of a 96-well qRT-PCR plate and mixed with 15 μl of H_2_O. Next, 2 μl of 100 μM DacA_CD_, DacA_CD_-C, DacA_CD_-K (monomer) or DacA_CD_-K (dimer) protein stock solutions were added and finally 1 μl of 100X Sypro orange was added to yield a total reaction volume of 20 μl and a final concentration of Sypro orange of 5X. The reactions for each protein were set up in triplicate in a 96-well plate. An Applied Biosystems OneStepPlus real-time PCR system was used for the thermal unfolding reactions and the temperature was increased every 30 s by 1°C from 25°C to 95°C and the fluorescence intensities measured. The data were analyzed using the Applied Biosystems StepOne Plus software. Fluorescence readings of the triplicate samples were averaged and normalized for each protein as previously described [61] using the following equation: (F-F_min_)/(F_max_-F_min_) where F is the fluorescence value at each temperature, F_min_ is the average minimum fluorescence value detected for the each protein and F_max_ the average maximum value detected for the each protein.

### Small-angle X-ray scattering

SAXS data for the tag-less DacA_CD_ and GlmM proteins as well as the DacA_CD_/GlmM complex were collected at the B21 beamline at the Diamond Light Source (Didcot, UK) using an Agilent 1200 HPLC system equipped with a Superdex 200 5/150 column (GE Healthcare). Prior to data collection, the column was equilibrated with 30 mM Tris pH 7.5, 150 mM NaCl. 50 μl of 12.8 mg/ml DacA_CD_, 10 mg/ml GlmM or 15 mg/ml DacA_CD_/GlmM protein solutions were loaded onto the size exclusion column and the data collected continuously during the sample elution. All datasets were analyzed using ScÅtter (http://www.bioisis.net) using the scattering frames corresponding to the elution peaks. Particle reconstruction was performed using DAMMIF [62], averaging 13 models in slow mode. Mean NSD values obtained from DAMFILT were 0.723 for the DacA_CD_/GlmM complex, 0.763 for GlmM and 0.738 for DacA_CD_.

### Quantification of c-di-AMP by ELISA

*E*. *coli* XL1-Blue strains containing plasmid pBAD33 and derivatives thereof allowing expression of the *S*. *aureus* DacA, YbbR and/or GlmM protein from an arabinose inducible promoter were grown overnight at 37°C in 5 ml LB + 0.2% glucose + 20 μg/ml chloramphenicol. The next day, the cultures were diluted 1:200 into 20 ml fresh LB medium supplemented with 20 μg/ml of chloramphenicol and incubated for 2 h 45 min at 37°C. Next, arabinose was added to a final concentration of 0.2% and the cultures incubated for a further 3 h at 37°C. The A_600_ was measured and cells equivalent to 10 ml at A_600_ of 1 were collected by centrifugation, washed twice with phosphate buffered saline (PBS) pH 7.4 and frozen. For the co-expression of the *S*. *aureus* DacA protein and the GlmM-His proteins from different bacteria, *E*. *coli* XL1-Blue strains containing plasmid pBAD33-*dacA* and the empty vector p*Trc*His60, or plasmids pMLJ4 (GlmM-His *E*. *coli*), pMLJ11 (GlmM-His *S*. *aureus*), or pMLD137 (GlmM-His *P*. *aeruginosa*) were grown overnight at 37°C in 5 ml LB + 0.2% glucose with 100 μg/ml ampicillin and 20 μg/ml chloramphenicol. The next day, the cultures were diluted 1:200 into 20 ml fresh LB medium supplemented with the appropriate antibiotics and incubated for 2 h 45 min at 37°C. Next, IPTG was added to a final concentration as indicated in the figure and legend and the cultures were incubated for 15 min at 37°C. Next, arabinose was added to a final concentration of 0.2% and the cultures incubated for a further 3 h at 37°C. The A_600_ was measured and cells equivalent to 10 ml of A_600_ of 1 were collected by centrifugation, the cells washed twice with phosphate buffered saline (PBS) pH 7.4 and frozen. For the ELISA assay, the cell pellets were thawed and suspended in 1 ml 50 mM Tris pH 8 buffer or 1 ml 50 mM Tris pH 8 buffer containing 1 mg/ml lysozyme. The cells were lysed by sonication for 2 to 3 min with 10 sec. on and 10 sec. off cycles at 40% amplitude and the samples were subsequently boiled for 10 min. The cell debris was removed by centrifugation at 20000 × g for 5 min and the soluble fraction transferred to a fresh tube. A competitive ELISA for the quantification of the cellular c-di-AMP levels was performed as previously described [12, 63]. Briefly, the ELISA was performed as follows: the wells of a NUNC Maxisorb ELISA 96-well plate were coated with the *Streptococcus pneumoniae* c-di-AMP binding protein CpaA by placing 100 μl of a 10 μg/ml protein solution in 50 mM Na_2_CO_3_, 50 mM NaHCO_3_ pH 9.6 buffer into the appropriate wells and the plate was subsequently incubated overnight at 4°C. The coating solution was removed, the wells washed 3 times with 200 μl PBS pH 7.4 containing 0.05% Tween 20 (PBST) and then blocked with 150 μl of 5% BSA in PBS pH 7.4 solution and the plate was incubated at 18°C for 1 h. Next, the blocking solution was removed and each well was washed 3 times with 200 μl PBST. Reference samples for the c-di-AMP concentration standard curve and the *E*. *coli* samples were prepared as follows: For the standard curve, c-di-AMP (Biolog) solutions in 50 mM Tris pH 8 buffer were prepared ranging from 300 nM to 3.125 μM and mixed with an equal volume of a 50 nM biotin-c-di-AMP solution. For the *E*. *coli* samples, appropriate dilutions of the extracts were prepared in 50 mM Tris pH 8 and each sample mixed with an equal volume of the 50 nM biotin c-di-AMP solution. Next, 100 μl of the standard solutions and *E*. *coli* samples were added in triplicate to the CpaA protein-coated ELISA plate. The plate was incubated at 18°C for 2 h and the wells were subsequently washed 3 times with 200 μl PBST buffer. Next, 100 μl of High Sensitivity Streptavidin solution (ThermoFisher Scientific) diluted 1:5,000 in PBS was added to each well and the plate incubated at 18°C for 1 hr. The wells were washed 3 times with 200 μl PBST buffer and finally 100 μl of the developing solution, which was made up by dissolving a 10 mg *o*-phenylenediamine dihydrochloride tablet (OPD) (Merck) in 20 ml citrate buffer pH 5 buffer supplemented with 20 μl 30% H_2_O_2_, was added to each well. The plate was incubated at 18°C in the dark for 30 min and the reaction stopped by the addition of 100 to 200 μl 2 M H_2_SO_4_ to each well. The absorbance was subsequently measured at 480 nm and the readings for the c-di-AMP standards were used to generate a second order polynomial calibration curve and the quadratic equation was used to determine the c-di-AMP concentrations (in μM) per ml *E*. *coli* samples with A_600_ = 10. For each experiment, the average value of the triplicate sample on the ELISA plate was determined. The experiment was performed three times with independent *E*. *coli* samples and as a final result the average value and standard deviations from all three experiments was plotted.

### Detection of DacA and YbbR by western blot and GlmM by coomassie staining

The *E*. *coli* cultures used for the detection of c-di-AMP by ELISA were also used for the detection of DacA and YbbR by western blot and GlmM by Coomassie staining or GlmM-His by western blot with an His antibody. Following the 3 h induction step with 0.2% arabinose, bacteria from 1-ml culture equivalent to an A_600_ of 1 were harvested and suspended in 100 μl 2× SDS sample buffer. The samples were boiled for 10 min and centrifuged for 5 min at 13,000 × g. Next, 10 μl of each sample was run on a 12% SDS-PAGE gel and to assess the overproduction of GlmM, proteins were visualized by Coomassie staining. The DacA and YbbR proteins were detected by western blot using protein-specific antibodies primary antibodies produced at Covalab and previously used [12, 45] and the GlmM-His proteins using an HRP-conjugated His-tag antibody (Sigma). To this end, the proteins were electro-transferred to PVDF membranes, the membranes blocked with 5% (w/v) milk in Tris buffered saline containing 0.1% (v/v) Tween-20 (TBST). The membranes were subsequently incubated with DacA or YbbR-specific polyclonal rabbit antibodies in 5% (w/v) milk in TBST buffer at a 1:5000 dilution. After washing the membranes with TBST buffer, they were incubated with the secondary HRP-conjugated anti-rabbit IgG antibody (Cell signaling Technology) used at a 1:10000 dilution. The HRP-linked His-tag antibody was used at a 1:5,000 or 1:10,000 dilution. Next, the membranes were washed and the blots developed using the Clarity Western ECL Blotting Substrate (Bio-Rad) and imaged using a ChemiDoc Touch system (Bio-Rad). The experiment was performed on three independently grown samples and a representative result is shown.

### Accession numbers

Structure coordinates were deposited in the Protein Database, under PDB codes 6GYW (nucleotide-free DacA_CD_), 6GYX (ApCpp-bound DacA_CD_), 6GYY (DacA_CD_-C mutant), 6GYZ (GlmM). SAXS models were deposited in the SASBDB database, under accession codes SASDE78 (DacA_CD_/GlmM complex), SASDE88 (GlmM) and SASDE98 (DacA_CD_).

## Supporting information

S1 Fig Model of the DacA_CD_ oligomerization required for catalytic activity. (A) Symmetry-related molecules found in the ApCpp-DacA_CD_ crystal lattice. Two DacA_CD_ dimers can be found close to each other, with the two incoming protomers not blocked by steric hindrance and free to be engaged in ATP condensation. ApCpp molecules are colored in yellow and shown as stick representations. (B) Model of c-di-AMP production by two interacting DacA_CD_ dimers. Two protomers can be engaged in a head-to-head transient dimer similar to that found in the catalytic domain of DisA (green, PDB 4YXJ), thus allowing the condensation of two ATP molecules.
(TIF)

S2 Fig Size exclusion profiles of WT DacA_CD_ and DacA_CD_ variants. WT DacA_CD_, DacA_CD_-C and DacA_CD_-K proteins were purified over a Ni-NTA column, the His-tags removed by thrombin cleavage and the proteins subsequently analyzed on a Superdex 200 10/300 size exclusion column and UV profiles recorded at 280 nm. The WT DacA_CD_ UV profile is shown in black, the DacA_CD_-K profile in blue and the DacA_CD_-C profile in red. The experiment was performed in duplicate and a representative result is shown.
(TIF)

S3 Fig Pull-down assay of GlmM-His and tag-less DacA_CD_. Coomassie-stained gel with fraction from an affinity pull-down experiment. Equimolar amounts of the GlmM-His and the tag-less DacA_CD_ protein were mixed and purified over a Ni-NTA column. Aliquots of the load, flow-through, wash and elution fractions were separated on 12% SDS-PAGE gel and proteins visualized by Coomassie staining. The experiment was performed in triplicate and a representative result is shown. As control, the tag-less DacA_CD_ protein was purified once over a Ni-NTA column in the absence of GlmM-His and load, flow-through, wash and elution fractions were analyzed on a 12% SDS-PAGE gel and proteins visualized by Coomassie staining.
(TIF)

S4 Fig SEC-MALS analysis of the purified tag-less DacA_CD_/GlmM complex. 100 μl of the purified, tag-less DacA_CD_/GlmM protein complex at 18 mg/ml were separated on a Superdex 200 Increase 10/300 column coupled to a MALS detector and refractometer. The UV absorbance, laser scattering and refractive index change were monitored. The data were analyzed using the ASTRA 6.0 software and fitted according to the Zimm model for static light scattering. The experiment was performed twice and a representative result is shown.
(TIF)

S5 Fig Expression of full-length DacA, YbbR and GlmM proteins in *E*. *coli*. *E*. *coli* strains containing pBAD33-derived vectors were grown to mid-log phase and expression of *dacA*, *dacA-ybbR*, *dacA-ybbR-glmM* or *dacA-no-ybbR-glmM* induced for 3 h by the addition of 0.2% arabinose. Subsequently, samples were prepared and proteins separated on 12% PAA gels and the DacA and YbbR proteins detected by western-blot and GlmM detected by Coomassie staining. The experiment was performed three times and a representative western-blot or Coomassie-stained gel is shown.
(TIF)

S6 Fig SAXS scattering curves and SEC-SAXS elution profiles of the purified, tag-less DacA_CD_/GlmM, GlmM and DacA_CD_ proteins. 50 μl of (A) DacA_CD_, (B) GlmM or (C) the DacA_CD_/GlmM complex were injected onto a Superdex 200 5/150 column coupled to the B21 Small-Angle X-Ray Beamline at Diamond Light Source (Didcot, UK). A full dataset of 620 scattering frames was collected and the data were analyzed with ScÅtter to calculate the scattering curves. (D) Radius of gyration (Rg) plots of DacA_CD_, GlmM and the DacA_CD_/GlmM complex were produced using the program ScÅtter. Scattering frames were selected according to homogeneity of the estimated Rg values.
(TIF)

S7 Fig Guinier plots and FOXS fitting curves of DacA_CD_, GlmM and the DacA_CD_/GlmM complex. Guinier plots (left) of (A) DacA_CD_, (B) GlmM or (C) the DacA_CD_/GlmM complex were analyzed using the program ScÅtter to assess sample homogeneity during the SAXS experiments. The structural models of DacA_CD_, GlmM and the complex were then used to calculate theoretical SAXS scattering curves using the program FOXS and subsequently compared to the experimental SAXS scattering curves. Fitting profiles of the experimental and theoretical curves are shown in the panels on the right.
(TIF)

S8 Fig Structure-based alignment of the different DAC domains. A structure-based alignment of the DAC domains from the *S*. *aureus* DacA_CD_ (DacA_Sau) starting from residue Y110 and ending with residue G260 (using full-length DacA amino acid numbering), *L*. *monocytogenes* CdaA_CD_ (CdaA_Lmo), *B*. *cereus* CdaS (CdaS_Bce), *T*. *maritima* DisA (DisA_Tma) was generated in STRAP. Conserved DGA and RHR motifs are highlighted in red. The position of the amino acid residue Y192 in the *S*. *aureus* DacA protein making an additional pi-stacking contact with the ribose base of the substrate is highlighted in teal. DacA_CD_ residues making contacts with the ApCpp ligand are highlighted with an asterisk.
(TIF)

S9 Fig Overlay of a DacA_CD_ dimer with the CdaS hexamer model. **The** CdaS hexamer model was built from symmetry mates of the CdaS trimer structure (PDB 2FB5), as reported in Mehne *et al*. [33]. The CdaS DAC domain is colored in green, while the two N-terminal helices are colored in purple. DacA_CD_ protomers are colored in cyan and brown. Active site residues are colored in black and red for CdaS and DacA_CD_, respectively. DacA_CD_ and CdaS DAC domain overlap with a r.m.s.d. of 0.58 Å.
(TIF)

S1 Table Crystallographic data and refinement statistics. (PDF)

S2 Table Theoretical and experimental masses of DacA_CD_ and GlmM species. (PDF)

S3 Table SAXS data statistics. (PDF)

S4 Table Collision Cross Section (CCS) parameters. (PDF)

S5 Table Bacterial strains used in this study. (PDF)

S6 Table Primers used in this study. (PDF)

## References

[1] Hengge R, Gründling A, Jenal U, Ryan R, Yildiz F. Bacterial Signal Transduction by Cyclic Di-GMP and Other Nucleotide Second Messengers. J Bacteriol. 2016;198(1):15–26. 10.1128/JB.00331-15 . 26055111 PMC4686208

[2] Corrigan RM, Campeotto I, Jeganathan T, Roelofs KG, Lee VT, Gründling A. Systematic identification of conserved bacterial c-di-AMP receptor proteins. Proc Natl Acad Sci U S A. 2013;110(22):9084–9. 10.1073/pnas.1300595110 . 23671116 PMC3670340

[3] Fahmi T, Port GC, Cho KH. c-di-AMP: An Essential Molecule in the Signaling Pathways that Regulate the Viability and Virulence of Gram-Positive Bacteria. Genes (Basel). 2017;8(8). 10.3390/genes8080197 . 28783096 PMC5575661

[4] Schuster CF, Bellows LE, Tosi T, Campeotto I, Corrigan RM, Freemont P, et al The second messenger c-di-AMP inhibits the osmolyte uptake system OpuC in Staphylococcus aureus. Sci Signal. 2016;9(441):ra81 10.1126/scisignal.aaf7279 . 27531650 PMC5248971

[5] Kim H, Youn SJ, Kim SO, Ko J, Lee JO, Choi BS. Structural Studies of Potassium Transport Protein KtrA Regulator of Conductance of K+ (RCK) C Domain in Complex with Cyclic Diadenosine Monophosphate (c-di-AMP). J Biol Chem. 2015;290(26):16393–402. 10.1074/jbc.M115.641340 . 25957408 PMC4481236

[6] Devaux L, Sleiman D, Mazzuoli MV, Gominet M, Lanotte P, Trieu-Cuot P, et al Cyclic di-AMP regulation of osmotic homeostasis is essential in Group B *Streptococcus*. PLoS Genet. 2018;14(4):e1007342 10.1371/journal.pgen.1007342 . 29659565 PMC5919688

[7] Bai Y, Yang J, Zarrella TM, Zhang Y, Metzger DW, Bai G. Cyclic di-AMP impairs potassium uptake mediated by a cyclic di-AMP binding protein in *Streptococcus pneumoniae*. J Bacteriol. 2014;196(3):614–23. 10.1128/JB.01041-13 . 24272783 PMC3911161

[8] Huynh TN, Choi PH, Sureka K, Ledvina HE, Campillo J, Tong L, et al Cyclic di-AMP targets the cystathionine beta-synthase domain of the osmolyte transporter OpuC. Mol Microbiol. 2016;102(2):233–43. 10.1111/mmi.13456 . 27378384 PMC5118871

[9] Commichau FM, Gibhardt J, Halbedel S, Gundlach J, Stulke J. A Delicate Connection: c-di-AMP Affects Cell Integrity by Controlling Osmolyte Transport. Trends in microbiology. 2018;26(3):175–85. Epub 2017/10/03. 10.1016/j.tim.2017.09.003 . 28965724

[10] Witte CE, Whiteley AT, Burke TP, Sauer JD, Portnoy DA, Woodward JJ. Cyclic di-AMP is critical for *Listeria monocytogenes* growth, cell wall homeostasis, and establishment of infection. MBio. 2013;4(3):e00282–13. 10.1128/mBio.00282-13 . 23716572 PMC3663569

[11] Whiteley AT, Garelis NE, Peterson BN, Choi PH, Tong L, Woodward JJ, et al c-di-AMP modulates *Listeria monocytogenes* central metabolism to regulate growth, antibiotic resistance and osmoregulation. Mol Microbiol. 2017;104(2):212–33. 10.1111/mmi.13622 . 28097715 PMC5391996

[12] Bowman L, Zeden MS, Schuster CF, Kaever V, Gründling A. New Insights into the Cyclic Di-adenosine Monophosphate (c-di-AMP) Degradation Pathway and the Requirement of the Cyclic Dinucleotide for Acid Stress Resistance in *Staphylococcus aureus*. J Biol Chem. 2016;291(53):26970–86. 10.1074/jbc.M116.747709 . 27834680 PMC5207132

[13] Dengler V, McCallum N, Kiefer P, Christen P, Patrignani A, Vorholt JA, et al Mutation in the c-di-AMP cyclase *dacA* affects fitness and resistance of methicillin resistant *Staphylococcus aureus*. PLoS One. 2013;8(8):e73512 10.1371/journal.pone.0073512 . 24013956 PMC3754961

[14] Griffiths JM, O’Neill AJ. Loss of function of the gdpP protein leads to joint beta-lactam/glycopeptide tolerance in *Staphylococcus aureus*. Antimicrob Agents Chemother. 2012;56(1):579–81. 10.1128/AAC.05148-11 . 21986827 PMC3256080

[15] Luo Y, Helmann JD. Analysis of the role of *Bacillus subtilis* sigma(M) in beta-lactam resistance reveals an essential role for c-di-AMP in peptidoglycan homeostasis. Mol Microbiol. 2012;83(3):623–39. Epub 2012/01/04. 10.1111/j.1365-2958.2011.07953.x . 22211522 PMC3306796

[16] Karinou E, Schuster CF, Pazos M, Vollmer W, Gründling A. Inactivation of the monofunctional peptidoglycan glycosyltransferase SgtB allows *Staphylococcus aureus* to survive in the absence of lipoteichoic acid. J Bacteriol. 2018 Epub 2018/10/17. 10.1128/jb.00574-18 . 30322854 PMC6287468

[17] Zhu Y, Pham TH, Nhiep TH, Vu NM, Marcellin E, Chakrabortti A, et al Cyclic-di-AMP synthesis by the diadenylate cyclase CdaA is modulated by the peptidoglycan biosynthesis enzyme GlmM in *Lactococcus lactis*. Mol Microbiol. 2016;99(6):1015–27. 10.1111/mmi.13281 . 26585449

[18] Gundlach J, Mehne FM, Herzberg C, Kampf J, Valerius O, Kaever V, et al An Essential Poison: Synthesis and Degradation of Cyclic Di-AMP in Bacillus subtilis. J Bacteriol. 2015;197(20):3265–74. 10.1128/JB.00564-15 . 26240071 PMC4573722

[19] Corrigan RM, Abbott JC, Burhenne H, Kaever V, Gründling A. c-di-AMP is a new second messenger in *Staphylococcus aureus* with a role in controlling cell size and envelope stress. PLoS Pathog. 2011;7(9):e1002217 10.1371/journal.ppat.1002217 . 21909268 PMC3164647

[20] Zeden MS, Schuster CF, Bowman L, Zhong Q, Williams HD, Gründling A. Cyclic di-adenosine monophosphate (c-di-AMP) is required for osmotic regulation in *Staphylococcus aureus* but dispensable for viability in anaerobic conditions. J Biol Chem. 2018;293(9):3180–200. 10.1074/jbc.M117.818716 . 29326168 PMC5836111

[21] Whiteley AT, Pollock AJ, Portnoy DA. The PAMP c-di-AMP Is Essential for *Listeria monocytogenes* Growth in Rich but Not Minimal Media due to a Toxic Increase in (p)ppGpp. Cell Host Microbe. 2015;17(6):788–98. 10.1016/j.chom.2015.05.006 . 26028365 PMC4469362

[22] Gundlach J, Herzberg C, Kaever V, Gunka K, Hoffmann T, Weiss M, et al Control of potassium homeostasis is an essential function of the second messenger cyclic di-AMP in *Bacillus subtilis*. Sci Signal. 2017;10(475). Epub 2017/04/20. 10.1126/scisignal.aal3011 . 28420751

[23] Witte G, Hartung S, Buttner K, Hopfner KP. Structural biochemistry of a bacterial checkpoint protein reveals diadenylate cyclase activity regulated by DNA recombination intermediates. Mol Cell. 2008;30(2):167–78. 10.1016/j.molcel.2008.02.020 . 18439896

[24] Corrigan RM, Gründling A. Cyclic di-AMP: another second messenger enters the fray. Nat Rev Microbiol. 2013;11(8):513–24. Epub 2013/07/03. 10.1038/nrmicro3069 . 23812326

[25] Rao F, See RY, Zhang D, Toh DC, Ji Q, Liang ZX. YybT is a signaling protein that contains a cyclic dinucleotide phosphodiesterase domain and a GGDEF domain with ATPase activity. J Biol Chem. 2010;285(1):473–82. Epub 2009/11/11. 10.1074/jbc.M109.040238 . 19901023 PMC2804195

[26] Huynh TN, Luo S, Pensinger D, Sauer JD, Tong L, Woodward JJ. An HD-domain phosphodiesterase mediates cooperative hydrolysis of c-di-AMP to affect bacterial growth and virulence. Proc Natl Acad Sci U S A. 2015;112(7):E747–56. 10.1073/pnas.1416485112 . 25583510 PMC4343097

[27] Tang Q, Luo Y, Zheng C, Yin K, Ali MK, Li X, et al Functional Analysis of a c-di-AMP-specific Phosphodiesterase MsPDE from *Mycobacterium smegmatis*. International journal of biological sciences. 2015;11(7):813–24. Epub 2015/06/17. 10.7150/ijbs.11797 . 26078723 PMC4466462

[28] Commichau FM, Heidemann JL, Ficner R, Stulke J. Making and breaking of an essential poison: the cyclases and phosphodiesterases that produce and degrade the essential second messenger cyclic di-AMP in bacteria. J Bacteriol. 2018 Epub 2018/09/19. 10.1128/jb.00462-18 . 30224435 PMC6287462

[29] Woodward JJ, Iavarone AT, Portnoy DA. c-di-AMP secreted by intracellular *Listeria monocytogenes* activates a host type I interferon response. Science. 2010;328(5986):1703–5. Epub 2010/05/29. 10.1126/science.1189801 . 20508090 PMC3156580

[30] Müller M, Deimling T, Hopfner KP, Witte G. Structural analysis of the diadenylate cyclase reaction of DNA-integrity scanning protein A (DisA) and its inhibition by 3'-dATP. Biochem J. 2015;469(3):367–74. 10.1042/BJ20150373 . 26014055

[31] Oppenheimer-Shaanan Y, Wexselblatt E, Katzhendler J, Yavin E, Ben-Yehuda S. c-di-AMP reports DNA integrity during sporulation in *Bacillus subtilis*. EMBO Rep. 2011;12(6):594–601. 10.1038/embor.2011.77 . 21566650 PMC3128283

[32] Zhang L, He ZG. Radiation-sensitive gene A (RadA) targets DisA, DNA integrity scanning protein A, to negatively affect cyclic Di-AMP synthesis activity in *Mycobacterium smegmatis*. J Biol Chem. 2013;288(31):22426–36. Epub 2013/06/14. 10.1074/jbc.M113.464883 . 23760274 PMC3829332

[33] Mehne FM, Schröder-Tittmann K, Eijlander RT, Herzberg C, Hewitt L, Kaever V, et al Control of the diadenylate cyclase CdaS in *Bacillus subtilis*: an autoinhibitory domain limits cyclic di-AMP production. J Biol Chem. 2014;289(30):21098–107. 10.1074/jbc.M114.562066 . 24939848 PMC4110313

[34] Zheng C, Ma Y, Wang X, Xie Y, Ali MK, He J. Functional analysis of the sporulation-specific diadenylate cyclase CdaS in *Bacillus thuringiensis*. Front Microbiol. 2015;6:908 10.3389/fmicb.2015.00908 . 26441857 PMC4568413

[35] Rosenberg J, Dickmanns A, Neumann P, Gunka K, Arens J, Kaever V, et al Structural and biochemical analysis of the essential diadenylate cyclase CdaA from *Listeria monocytogenes*. J Biol Chem. 2015;290(10):6596–606. 10.1074/jbc.M114.630418 . 25605729 PMC4358292

[36] Mengin-Lecreulx D, van Heijenoort J. Characterization of the essential gene glmM encoding phosphoglucosamine mutase in *Escherichia coli*. J Biol Chem. 1996;271(1):32–9. . 8550580 10.1074/jbc.271.1.32

[37] Levin S, Almo SC, Satir BH. Functional diversity of the phosphoglucomutase superfamily: structural implications. Protein Eng. 1999;12(9):737–46. . 10506283 10.1093/protein/12.9.737

[38] Pham TH, Liang ZX, Marcellin E, Turner MS. Replenishing the cyclic-di-AMP pool: regulation of diadenylate cyclase activity in bacteria. Curr Genet. 2016;62(4):731–8. 10.1007/s00294-016-0600-8 . 27074767

[39] Paxman JJ, Heras B. Bioinformatics Tools and Resources for Analyzing Protein Structures. Methods Mol Biol. 2017;1549:209–20. 10.1007/978-1-4939-6740-7_16 . 27975294

[40] Mengin-Lecreulx D, van Heijenoort J. Copurification of glucosamine-1-phosphate acetyltransferase and N-acetylglucosamine-1-phosphate uridyltransferase activities of *Escherichia coli*: characterization of the glmU gene product as a bifunctional enzyme catalyzing two subsequent steps in the pathway for UDP-N-acetylglucosamine synthesis. J Bacteriol. 1994;176(18):5788–95. . 8083170 10.1128/jb.176.18.5788-5795.1994 PMC196783

[41] Mehra-Chaudhary R, Mick J, Beamer LJ. Crystal structure of *Bacillus anthracis* phosphoglucosamine mutase, an enzyme in the peptidoglycan biosynthetic pathway. J Bacteriol. 2011;193(16):4081–7. 10.1128/JB.00418-11 . 21685296 PMC3147701

[42] Mehra-Chaudhary R, Mick J, Tanner JJ, Beamer LJ. Quaternary structure, conformational variability and global motions of phosphoglucosamine mutase. FEBS J. 2011;278(18):3298–307. 10.1111/j.1742-4658.2011.08246.x . 21767345

[43] Pettersen EF, Goddard TD, Huang CC, Couch GS, Greenblatt DM, Meng EC, et al UCSF Chimera—a visualization system for exploratory research and analysis. J Comput Chem. 2004;25(13):1605–12. 10.1002/jcc.20084 . 15264254

[44] Degiacomi MT, Benesch JL. EM intersectionIM: software for relating ion mobility mass spectrometry and electron microscopy data. Analyst. 2016;141(1):70–5. 10.1039/c5an01636c . 26616427

[45] Corrigan RM, Bowman L, Willis AR, Kaever V, Gründling A. Cross-talk between two nucleotide-signaling pathways in *Staphylococcus aureus*. J Biol Chem. 2015;290(9):5826–39. 10.1074/jbc.M114.598300 . 25575594 PMC4342491

[46] Gille C, Frommel C. STRAP: editor for STRuctural Alignments of Proteins. Bioinformatics. 2001;17(4):377–8. . 11301311 10.1093/bioinformatics/17.4.377

[47] Bai Y, Yang J, Zhou X, Ding X, Eisele LE, Bai G. *Mycobacterium tuberculosis* Rv3586 (DacA) is a diadenylate cyclase that converts ATP or ADP into c-di-AMP. PLoS One. 2012;7(4):e35206 10.1371/journal.pone.0035206 . 22529992 PMC3328451

[48] Mehne FM, Gunka K, Eilers H, Herzberg C, Kaever V, Stülke J. Cyclic di-AMP homeostasis in *Bacillus subtilis*: both lack and high level accumulation of the nucleotide are detrimental for cell growth. J Biol Chem. 2013;288(3):2004–17. Epub 2012/11/30. 10.1074/jbc.M112.395491 . 23192352 PMC3548507

[49] Glanzmann P, Gustafson J, Komatsuzawa H, Ohta K, Berger-Bächi B. *glmM* operon and methicillin-resistant *glmM* suppressor mutants in *Staphylococcus aureus*. Antimicrob Agents Chemother. 1999;43(2):240–5. Epub 1999/01/30. . 9925512 10.1128/aac.43.2.240 PMC89057

[50] Sureka K, Choi PH, Precit M, Delince M, Pensinger DA, Huynh TN, et al The cyclic dinucleotide c-di-AMP is an allosteric regulator of metabolic enzyme function. Cell. 2014;158(6):1389–401. 10.1016/j.cell.2014.07.046 . 25215494 PMC4166403

[51] Pringle SD, Giles K, Wildgoose JL, Williams JP, Slade SE, Thalassinos K, et al An investigation of the mobility separation of some peptide and protein ions using a new hybrid quadrupole/travelling wave IMS/oa-ToF instrument. International Journal of Mass Spectrometry. 2007;261(1):1–12. 10.1016/j.ijms.2006.07.021.

[52] Thalassinos K, Grabenauer M, Slade SE, Hilton GR, Bowers MT, Scrivens JH. Characterization of phosphorylated peptides using traveling wave-based and drift cell ion mobility mass spectrometry. Anal Chem. 2009;81(1):248–54. 10.1021/ac801916h . 19117454

[53] Sivalingam GN, Yan J, Sahota H, Thalassinos K. Amphitrite: A program for processing travelling wave ion mobility mass spectrometry data. Int J Mass Spectrom. 2013;345–347:54–62. 10.1016/j.ijms.2012.09.005 . 25844045 PMC4375678

[54] Winter G, Waterman DG, Parkhurst JM, Brewster AS, Gildea RJ, Gerstel M, et al DIALS: implementation and evaluation of a new integration package. Acta Crystallogr D Struct Biol. 2018;74(Pt 2):85–97. 10.1107/S2059798317017235 . 29533234 PMC5947772

[55] Evans PR, Murshudov GN. How good are my data and what is the resolution? Acta Crystallogr D Biol Crystallogr. 2013;69(Pt 7):1204–14. 10.1107/S0907444913000061 . 23793146 PMC3689523

[56] Evans PR. An introduction to data reduction: space-group determination, scaling and intensity statistics. Acta Crystallogr D Biol Crystallogr. 2011;67(Pt 4):282–92. 10.1107/S090744491003982X . 21460446 PMC3069743

[57] He H, Fang H, Miller MD, Phillips GN Jr., Su WP. Improving the efficiency of molecular replacement by utilizing a new iterative transform phasing algorithm. Acta Crystallogr A Found Adv. 2016;72(Pt 5):539–47. 10.1107/S2053273316010731 . 27580202 PMC5006650

[58] Zwart PH, Afonine PV, Grosse-Kunstleve RW, Hung LW, Ioerger TR, McCoy AJ, et al Automated structure solution with the PHENIX suite. Methods Mol Biol. 2008;426:419–35. 10.1007/978-1-60327-058-8_28 . 18542881

[59] Emsley P, Lohkamp B, Scott WG, Cowtan K. Features and development of Coot. Acta Crystallogr D Biol Crystallogr. 2010;66(Pt 4):486–501. 10.1107/S0907444910007493 . 20383002 PMC2852313

[60] Schrodinger, LLC. The PyMOL Molecular Graphics System, Version 1.7. 2015.

[61] Boivin S, Kozak S, Meijers R. Optimization of protein purification and characterization using Thermofluor screens. Protein Expr Purif. 2013;91(2):192–206. 10.1016/j.pep.2013.08.002 . 23948764

[62] Franke D, Svergun DI. DAMMIF, a program for rapid ab-initio shape determination in small-angle scattering. J Appl Crystallogr. 2009;42(Pt 2):342–6. 10.1107/S0021889809000338 . 27630371 PMC5023043

[63] Underwood AJ, Zhang Y, Metzger DW, Bai G. Detection of cyclic di-AMP using a competitive ELISA with a unique pneumococcal cyclic di-AMP binding protein. J Microbiol Methods. 2014;107:58–62. 10.1016/j.mimet.2014.08.026 . 25239824 PMC4252578

