# Supplementary figures and images for: Inhibition of the Staphylococcus aureus c-di-AMP cyclase DacA by direct interaction with the phosphoglucosamine mutase GlmM
Source: PLoS Pathog. 2019 Jan 22;15(1):e1007537. doi: 10.1371/journal.ppat.1007537 (PMC6368335; doi:10.1371/journal.ppat.1007537)

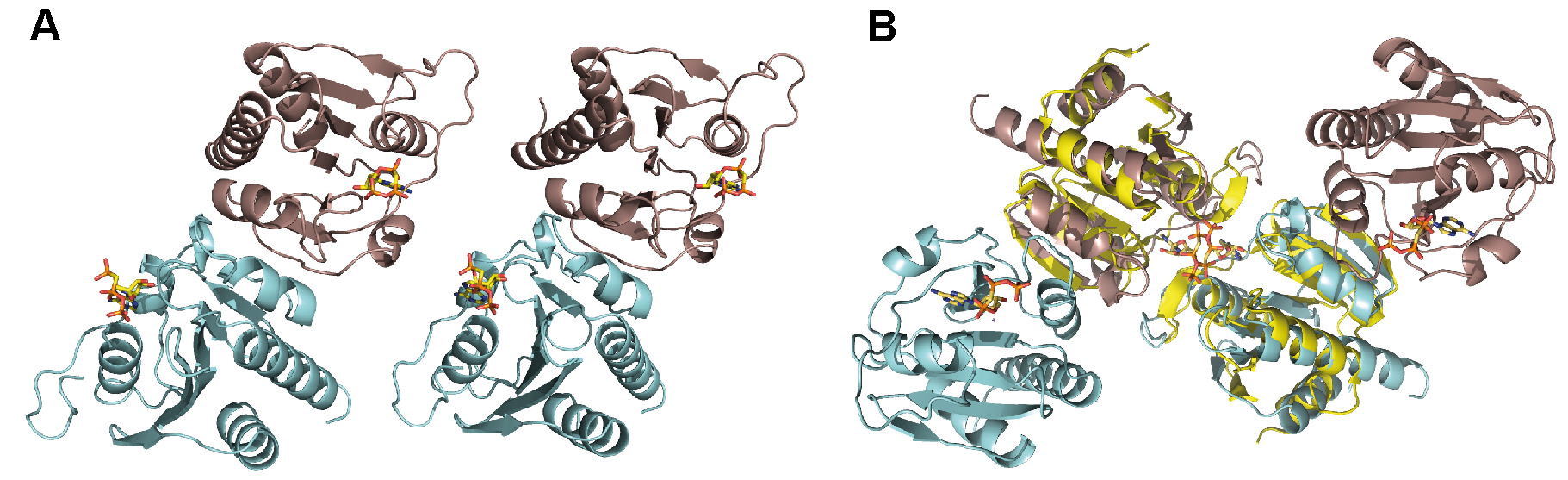

Supplement: S1 Fig — (A) Symmetry-related molecules found in the ApCpp-DacACD crystal lattice. Two DacACD dimers can be found close to each other, with the two incoming protomers not blocked by steric hindrance and free to be engaged in ATP condensation. ApCpp molecules are colored in yellow and shown as stick representations. (B) Model of c-di-AMP production by two interacting DacACD dimers. Two protomers can be engaged in a head-to-head transient dimer similar to that found in the catalytic domain of DisA (green, PDB 4YXJ), thus allowing the condensation of two ATP molecules. (TIF) [file ppat.1007537.s001.tif]

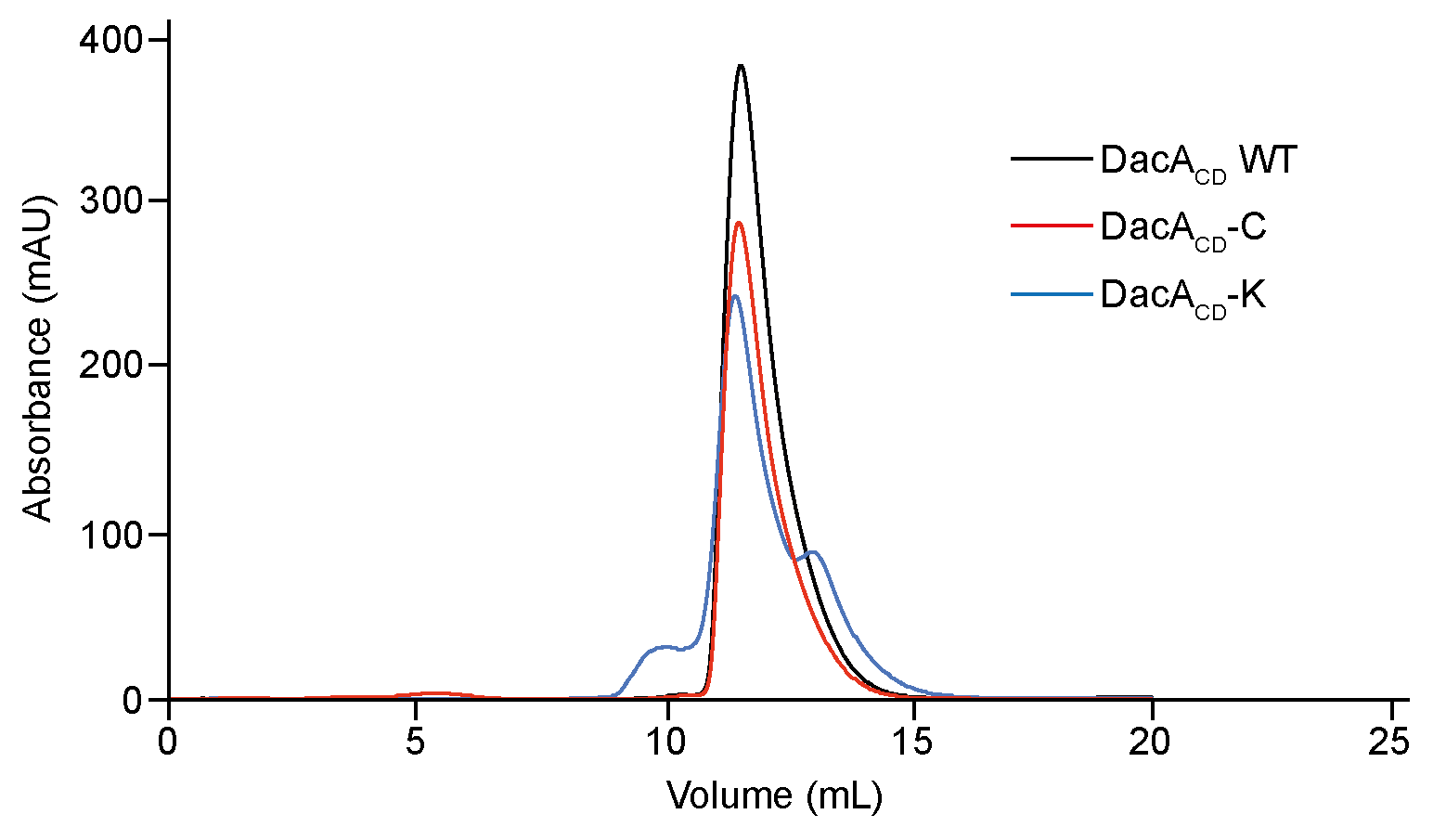

Supplement: S2 Fig — WT DacACD, DacACD-C and DacACD-K proteins were purified over a Ni-NTA column, the His-tags removed by thrombin cleavage and the proteins subsequently analyzed on a Superdex 200 10/300 size exclusion column and UV profiles recorded at 280 nm. The WT DacACD UV profile is shown in black, the DacACD-K profile in blue and the DacACD-C profile in red. The experiment was performed in duplicate and a representative result is shown. (TIF) [file ppat.1007537.s002.tif]

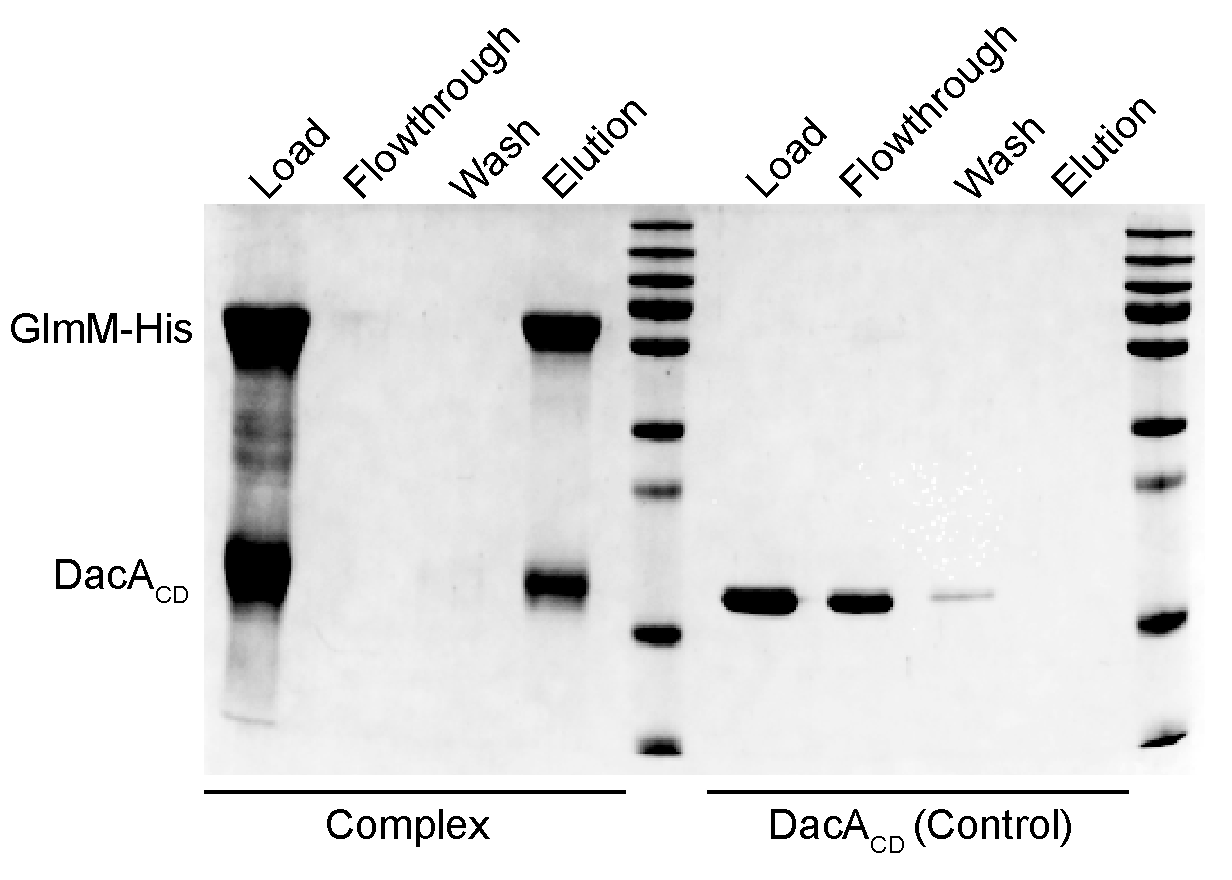

Supplement: S3 Fig — Coomassie-stained gel with fraction from an affinity pull-down experiment. Equimolar amounts of the GlmM-His and the tag-less DacACD protein were mixed and purified over a Ni-NTA column. Aliquots of the load, flow-through, wash and elution fractions were separated on 12% SDS-PAGE gel and proteins visualized by Coomassie staining. The experiment was performed in triplicate and a representative result is shown. As control, the tag-less DacACD protein was purified once over a Ni-NTA column in the absence of GlmM-His and load, flow-through, wash and elution fractions were analyzed on a 12% SDS-PAGE gel and proteins visualized by Coomassie staining. (TIF) [file ppat.1007537.s003.tif]

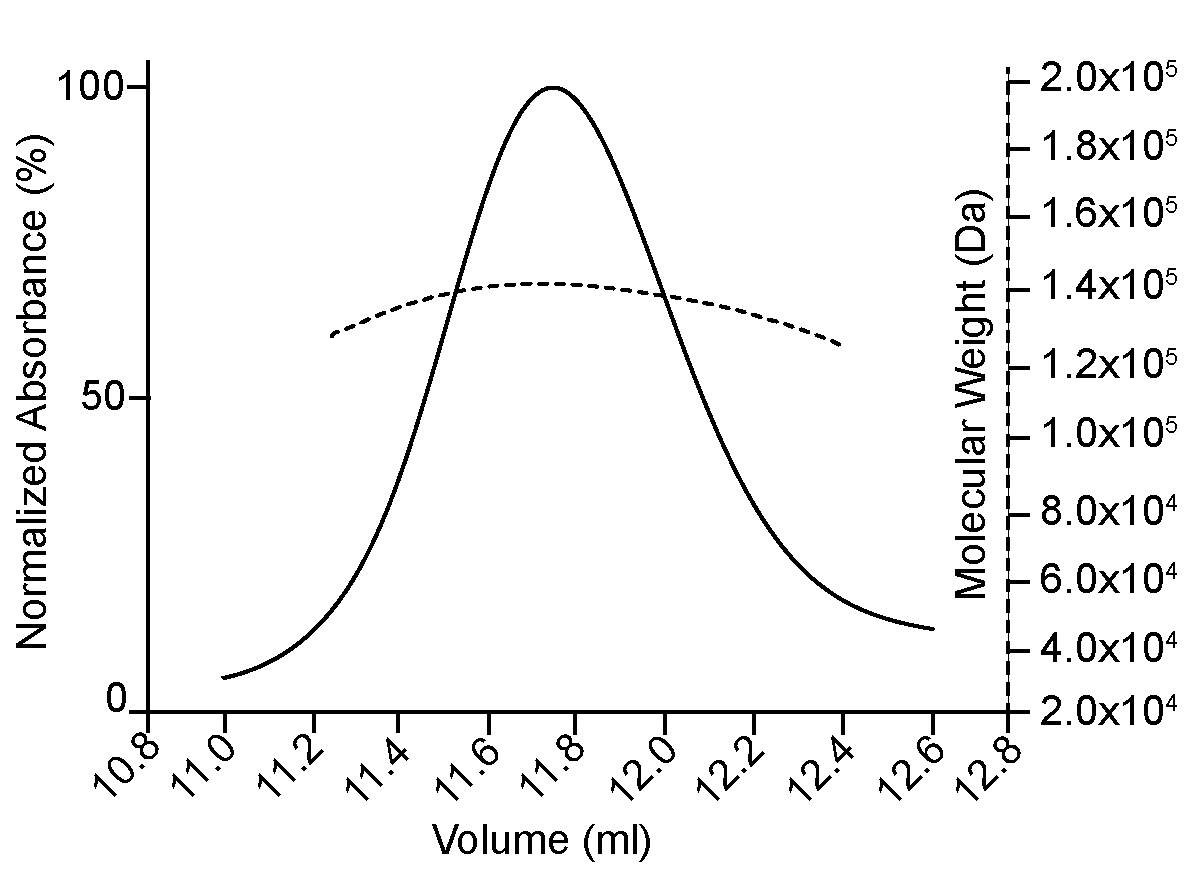

Supplement: S4 Fig — 100 μl of the purified, tag-less DacACD/GlmM protein complex at 18 mg/ml were separated on a Superdex 200 Increase 10/300 column coupled to a MALS detector and refractometer. The UV absorbance, laser scattering and refractive index change were monitored. The data were analyzed using the ASTRA 6.0 software and fitted according to the Zimm model for static light scattering. The experiment was performed twice and a representative result is shown. (TIF) [file ppat.1007537.s004.tif]

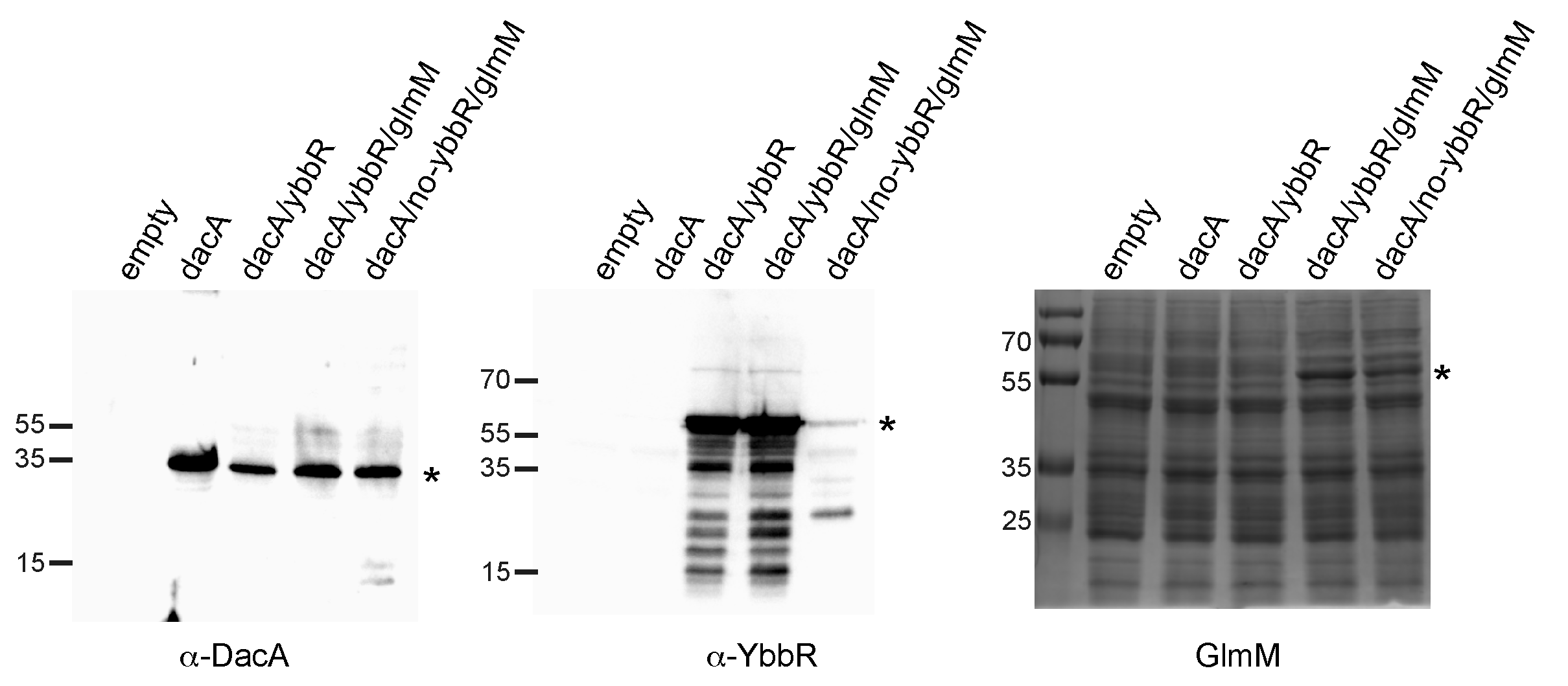

Supplement: S5 Fig — E. coli strains containing pBAD33-derived vectors were grown to mid-log phase and expression of dacA, dacA-ybbR, dacA-ybbR-glmM or dacA-no-ybbR-glmM induced for 3 h by the addition of 0.2% arabinose. Subsequently, samples were prepared and proteins separated on 12% PAA gels and the DacA and YbbR proteins detected by western-blot and GlmM detected by Coomassie staining. The experiment was performed three times and a representative western-blot or Coomassie-stained gel is shown. (TIF) [file ppat.1007537.s005.tif]

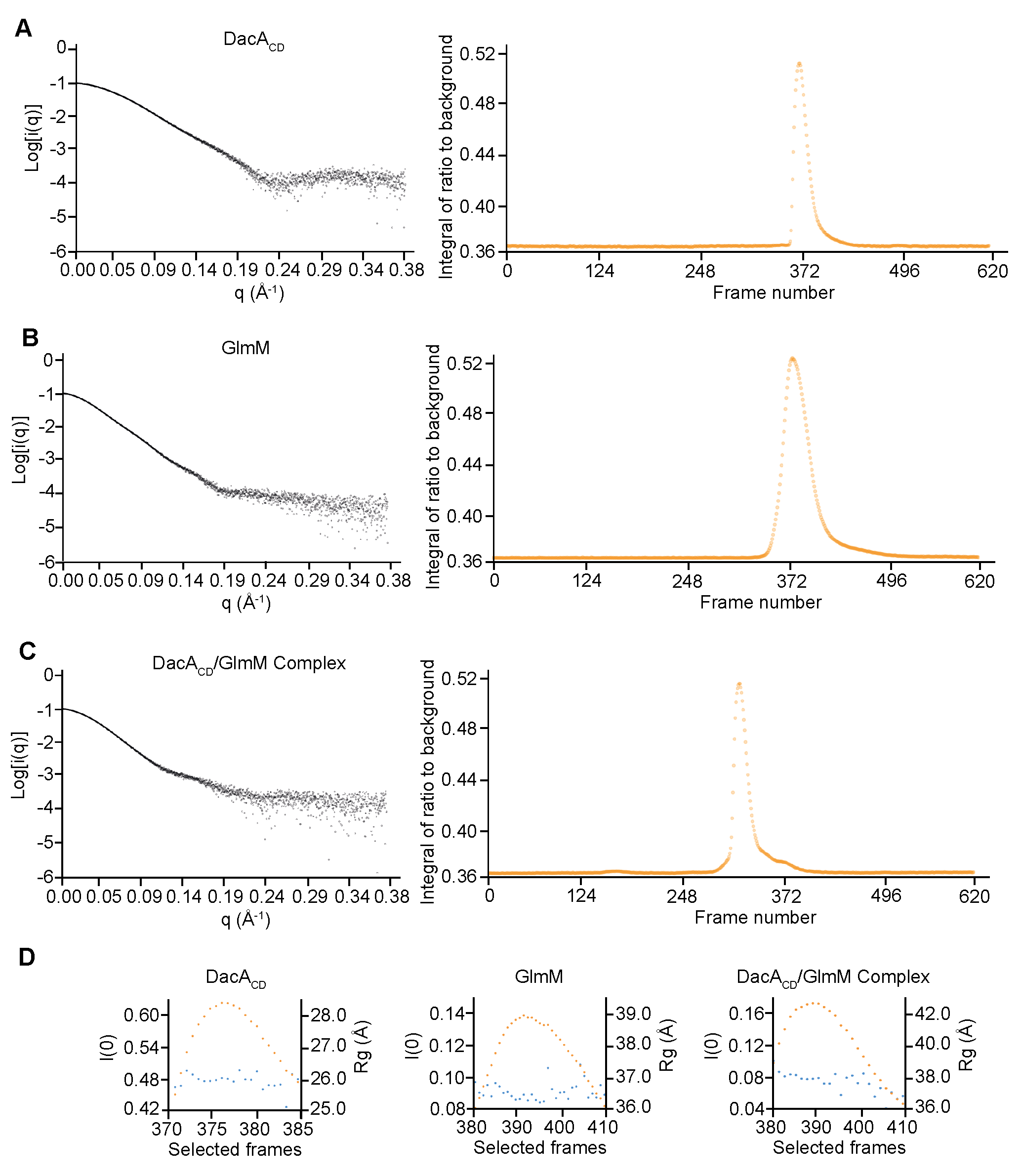

Supplement: S6 Fig — 50 μl of (A) DacACD, (B) GlmM or (C) the DacACD/GlmM complex were injected onto a Superdex 200 5/150 column coupled to the B21 Small-Angle X-Ray Beamline at Diamond Light Source (Didcot, UK). A full dataset of 620 scattering frames was collected and the data were analyzed with ScÅtter to calculate the scattering curves. (D) Radius of gyration (Rg) plots of DacACD, GlmM and the DacACD/GlmM complex were produced using the program ScÅtter. Scattering frames were selected according to homogeneity of the estimated Rg values. (TIF) [file ppat.1007537.s006.tif]

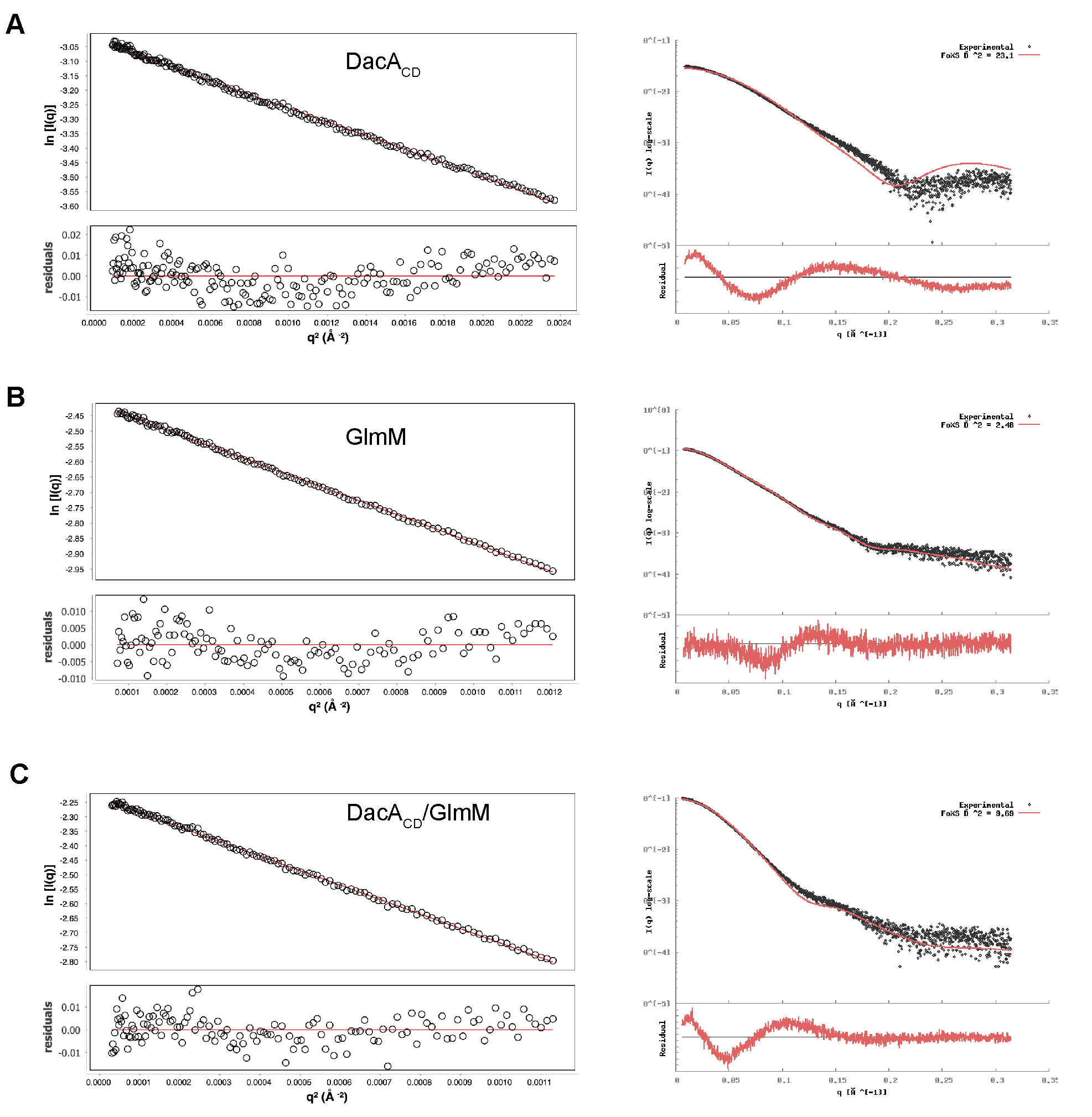

Supplement: S7 Fig — Guinier plots (left) of (A) DacACD, (B) GlmM or (C) the DacACD/GlmM complex were analyzed using the program ScÅtter to assess sample homogeneity during the SAXS experiments. The structural models of DacACD, GlmM and the complex were then used to calculate theoretical SAXS scattering curves using the program FOXS and subsequently compared to the experimental SAXS scattering curves. Fitting profiles of the experimental and theoretical curves are shown in the panels on the right. (TIF) [file ppat.1007537.s007.tif]

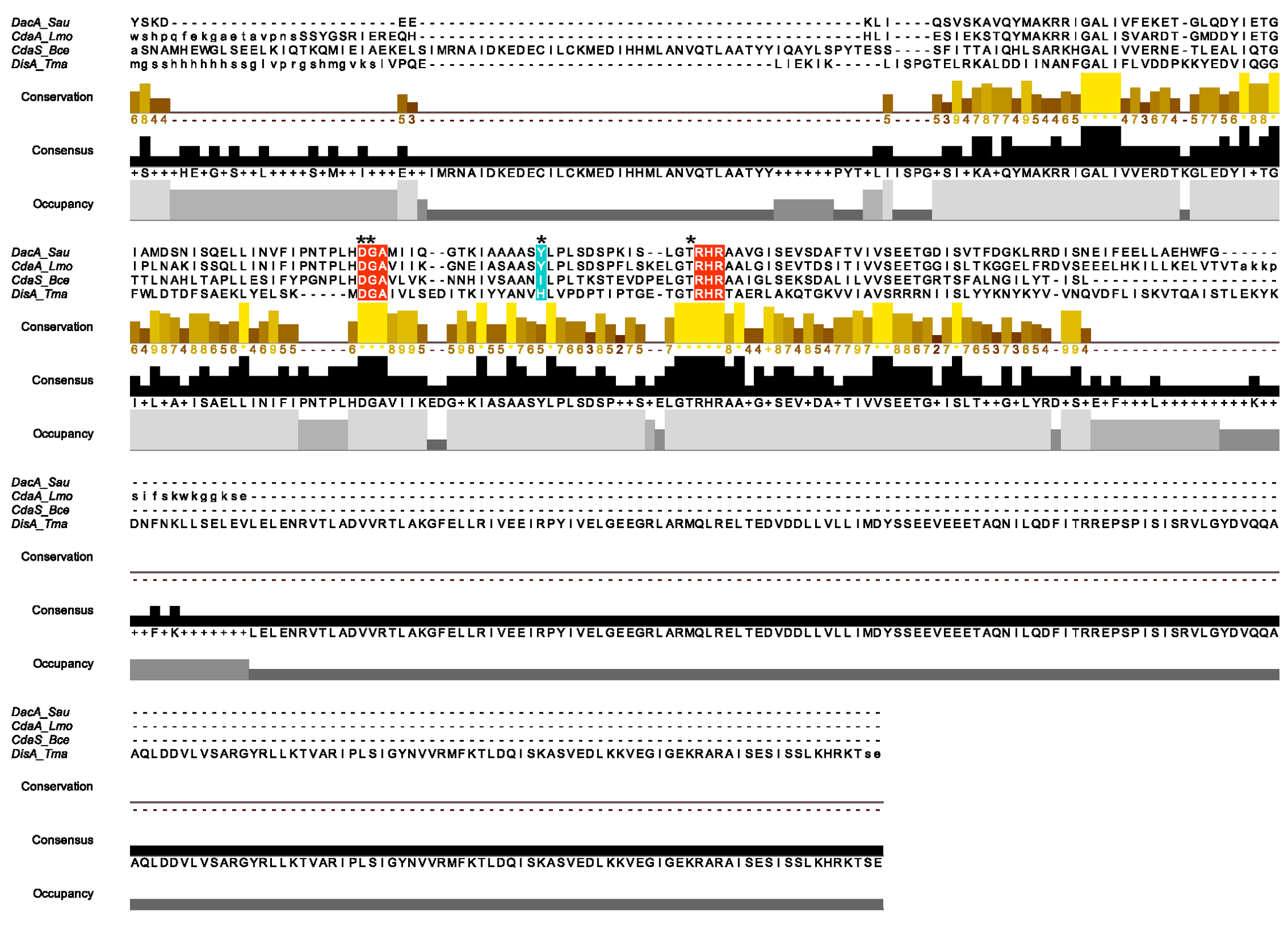

Supplement: S8 Fig — A structure-based alignment of the DAC domains from the S. aureus DacACD (DacA_Sau) starting from residue Y110 and ending with residue G260 (using full-length DacA amino acid numbering), L. monocytogenes CdaACD (CdaA_Lmo), B. cereus CdaS (CdaS_Bce), T. maritima DisA (DisA_Tma) was generated in STRAP. Conserved DGA and RHR motifs are highlighted in red. The position of the amino acid residue Y192 in the S. aureus DacA protein making an additional pi-stacking contact with the ribose base of the substrate is highlighted in teal. DacACD residues making contacts with the ApCpp ligand are highlighted with an asterisk. (TIF) [file ppat.1007537.s008.tif]

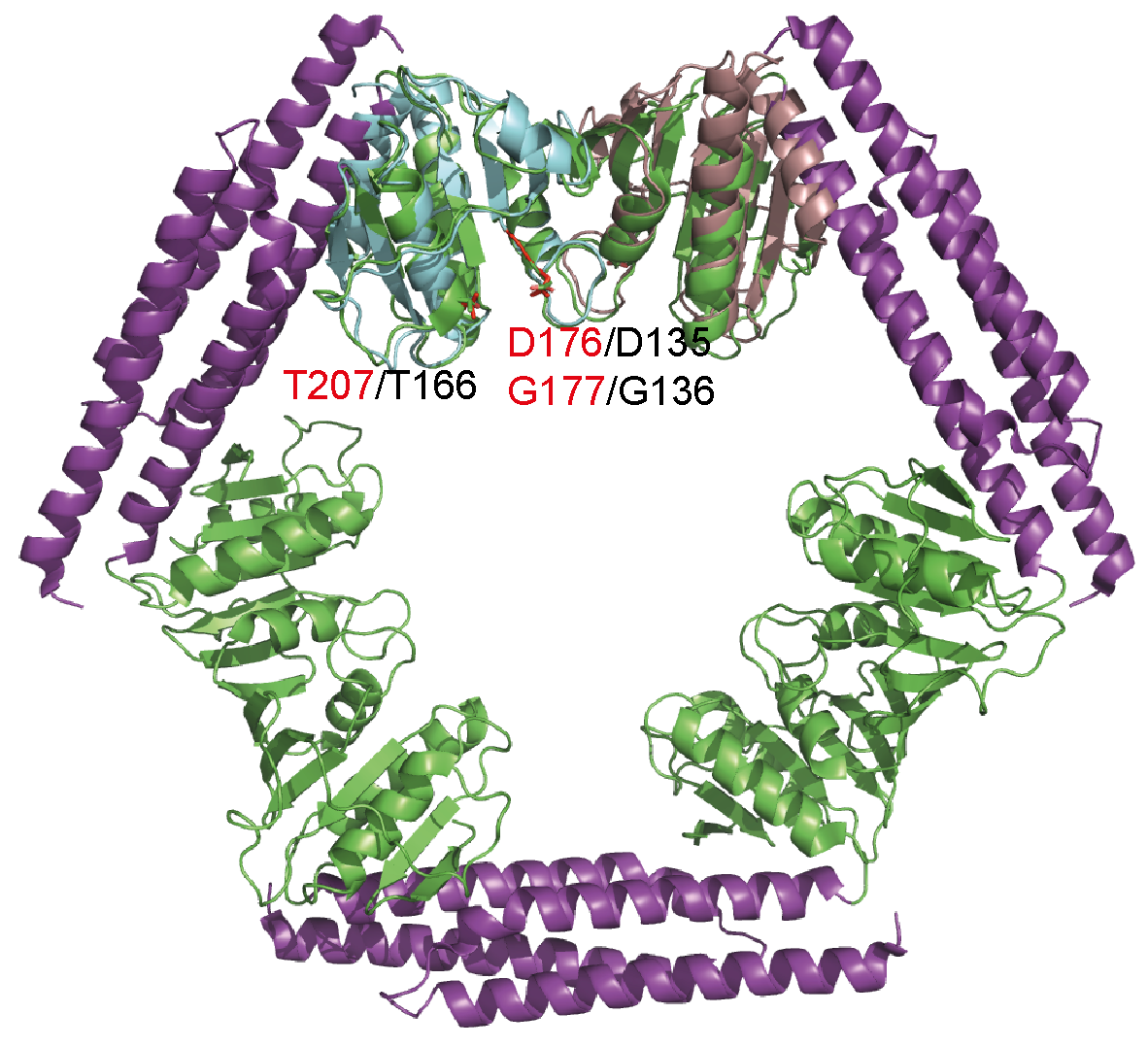

Supplement: S9 Fig — The CdaS hexamer model was built from symmetry mates of the CdaS trimer structure (PDB 2FB5), as reported in Mehne et al. [33]. The CdaS DAC domain is colored in green, while the two N-terminal helices are colored in purple. DacACD protomers are colored in cyan and brown. Active site residues are colored in black and red for CdaS and DacACD, respectively. DacACD and CdaS DAC domain overlap with a r.m.s.d. of 0.58 Å. (TIF) [file ppat.1007537.s009.tif]
