# Supplementary material for: Inhibition of the Staphylococcus aureus c-di-AMP cyclase DacA by direct interaction with the phosphoglucosamine mutase GlmM
Source: PLoS Pathog. 2019 Jan 22;15(1):e1007537. doi: 10.1371/journal.ppat.1007537 (PMC6368335; doi:10.1371/journal.ppat.1007537)
Supplement: S1 Table — (PDF) [file ppat.1007537.s010.pdf]

S1 Table: Crystallographic data and refinement statistics

	**DacA$_{CD}$ (apo)**	**DacA$_{CD}$ (ApCpp)**	**DacA$_{CD}$-C**	**GlmM**
**Data collection**				
Space group	P 21 21 21	P 21 21 21	P 21 21 21	P 21 21 21
Cell dimensions				
a, b, c (Å)	54.8 68.19 76.80	44.09 80.42 96.19	44.86 79.77 98.81	88.65 93.88 156.31
α, β, γ (˚)	90.00 90.00 90.00	90.00 90.00 90.00	90.00 90.00 90.00	90.00 90.00 90.00
Resolution (Å)	68.19 (1.7)*	61.70 (2.6)	98.81 (2.77)	93.88 (3.0)
R_{merge}	0.077 (0.627)	0.116 (2.115)	0.084 (1.147)	0.158 (1.868)
$CC_{(1/2)}$	0.999 (0.968)	0.997 (0.825)	0.998 (0.892)	0.998 (0.842)
I / σ(I)	13.9 (2.0)	10.2 (2.0)	14.2 (2.2)	8.4 (1.8)
Completeness (%)	99.9 (99.0)	99.3 (99.2)	99.9 (99.9)	100.0 (100.0)
Redundancy	12.2 (10.0)	12.2 (12.3)	12.0 (12.2)	12.5 (12.5)
**Refinement**				
Resolution (Å)	1.7	2.6	2.77	3.0
No. reflections	395176	133313	114145	334706
Unique reflections	32376	10930	9512	26871
R_{work} / R_{free}	0.1735/0.2004	0.2313/0.2576	0.2101/0.2312	0.2146/0.2547
R.m.s. deviations				
Bond lengths (Å)	0.006	0.004	0.008	0.004
Bond angles (˚)	0.962	0.731	1.199	0.789

* refers to the highest resolution shell
