# Supplementary material for: Inhibition of the Staphylococcus aureus c-di-AMP cyclase DacA by direct interaction with the phosphoglucosamine mutase GlmM
Source: PLoS Pathog. 2019 Jan 22;15(1):e1007537. doi: 10.1371/journal.ppat.1007537 (PMC6368335; doi:10.1371/journal.ppat.1007537)
Supplement: S2 Table — (PDF) [file ppat.1007537.s011.pdf]

S2 Table: Theoretical and calculated experimental masses of $DacA_{CD}$ and GlmM species

Species	Theoretical Mass (Da)	Calculated Mass (Da)
**Tetramer**	138,210	139,003 ± 10.41
**GlmM - dimer**	99,462	99,983 ± 19.86
**GlmM – monomer**	49,731	50,302 ± 22.99
**DacA – dimer**	38,748	39,232 ± 20.91
**DacA – monomer**	19,374	19,697 ± 59.15
**Octamer**	276,420	277,896 ± 111.24
