# Supplementary material for: Inhibition of the Staphylococcus aureus c-di-AMP cyclase DacA by direct interaction with the phosphoglucosamine mutase GlmM
Source: PLoS Pathog. 2019 Jan 22;15(1):e1007537. doi: 10.1371/journal.ppat.1007537 (PMC6368335; doi:10.1371/journal.ppat.1007537)
Supplement: S3 Table — (PDF) [file ppat.1007537.s012.pdf]

S3 Table: SAXS data statistics

	$DacA_{CD}$	GlmM	$DacA_{CD}$/GlmM
Beamline	B21	B21	B21
Wavelength (Å)	1.0	1.0	1.0
q-range (Å-1)	0.0032-0.38	0.0032-0.38	0.0032-0.38
I(0) (cm-1)	0.048 ± 4.4x10-5	0.09 ± 6.8x10-5	0.12 ± 1.2x10-4
Rg (Å)	26.3 ± 0.27	36.8 ± 0.26	38.7 ± 0.10
Dmax (Å)	85	121.5	125
Guinier region	0.2280-1.2928	0.2928-1.2840	0.2049-1.2919
Porod Volume (Å3)	64500	128000	204000
MW Estimate (kDa)	40.2	94.2	130.9
Number of atoms (Dammif)	1895	2491	2235
