# Supplementary material for: Inhibition of the Staphylococcus aureus c-di-AMP cyclase DacA by direct interaction with the phosphoglucosamine mutase GlmM
Source: PLoS Pathog. 2019 Jan 22;15(1):e1007537. doi: 10.1371/journal.ppat.1007537 (PMC6368335; doi:10.1371/journal.ppat.1007537)
Supplement: S4 Table — (PDF) [file ppat.1007537.s013.pdf]

S4 Table: Collision Cross section (CCS) parameters

	Theoretical CCS (Å^2)* IMPACT	EM∩IM	Charge State	Calculated $^{TW}CCS_{N2\rightarrow He2}$ (Å^2)
			21+	6690
**Tetramer**	-	6790	22+	6610
			23+	6460
			24+	6340
			18+	4750
**GlmM - Dimer**	6890	5550	19+	5220
			20+	5310
			21+	5420
**GlmM - Monomer**	3820	-	13+	3210
			14+	3350
**DacA - Dimer**	2870	3200	12+	2890
			13+	3080
**DacA-Monomer**	1620	-	8+	1810
			9+	1970

*IMPACT values refer to crystal structure data. EM∩IM values are based on SAXS structural models.
