# Supplementary material for: Inhibition of the Staphylococcus aureus c-di-AMP cyclase DacA by direct interaction with the phosphoglucosamine mutase GlmM
Source: PLoS Pathog. 2019 Jan 22;15(1):e1007537. doi: 10.1371/journal.ppat.1007537 (PMC6368335; doi:10.1371/journal.ppat.1007537)
Supplement: S5 Table — (PDF) [file ppat.1007537.s014.pdf]

S5 Table: Bacterial strains used in this study

Unique ID	Relevant features	Reference
	***Escherichia coli* strains**	
ANG127	XL1-Blue- Cloning strain; TetR –	Stratagene
ANG191	Protein expression strain –BL21(DE3)	Novagen
ANG1858	XL1-Blue pET28b-His-*dacA*$_{CD}$; KanR	This study
ANG1865	BL21 (DE3) pET28b-His-*dacA*$_{CD}$; KanR	This study
ANG3994	XL1-Blue pET28b-*glmM*-His; KanR	This study
ANG3997	BL21 (DE3) pET28b-*glmM*-His; KanR	This study
ANG4319	XL1-Blue pET28b-His-*dacA*$_{CD}$-K; KanR	This study
ANG4320	BL21 (DE3) pET28b-His-*dacA*$_{CD}$-K; KanR	This study
ANG4321	XL1-Blue pET28b-His-*dacA*$_{CD}$-C; KanR	This study
ANG4322	BL21 (DE3) pET28b-His-*dacA*$_{CD}$-C; KanR	This study
ANG4066	XL1-Blue pBAD33; CamR	[1]
ANG4120	XL1-Blue pBAD33-*dacA*; CamR	This study
ANG4121	XL1-Blue pBAD33-*dacA-ybbR*; CamR	This study
ANG4122	XL1-Blue pBAD33-*dacA-ybbR-glmM*; CamR	This study
ANG4263	XL1-Blue pBAD33-*dacA-no-ybbR-glmM*; CamR	This study
ANG5168	XL1-Blue p*Trc*His60	[2]
ANG5169	XL1-Blue pMLJ4 (GlmM-His *E. coli*), AmpR	[3]
ANG5171	XL1-Blue pMLJ11 (GlmM-His *S. aureus*), AmpR	Mengin-Lecreulx
ANG5172	XL1-Blue pMLD137 (GlmM-His *P. aeruginosa*), AmpR	[4]
ANG5174	XL1-Blue pBAD33-*dacA*, p*Trc*His60; CamR, AmpR	This study
ANG5175	XL1-Blue pBAD33-*dacA* pMLJ4 (GlmM-His *E. coli*), CamR, AmpR	This study
ANG5177	XL1-Blue pBAD33-*dacA* pMLJ11 (GlmM-His *S. aureus*), CamR, AmpR	This study
ANG5178	XL1-Blue pBAD33-*dacA* pMLD137 (GlmM-His *P. aeruginosa*), CamR, AmpR	This study
	Staphylococcus aureus strains	
AH1263	LAC* – ANG1575	[5]

Antibiotics were used at the following concentrations: Tetracycline (TetR) 10 μg/ml, Kanamycin (KanR) 30 μg/ml, Ampicillin (AmpR) 100 μg/ml, Chloramphenicol (CamR) 20 μg/ml

1. Guzman LM, Belin D, Carson MJ, Beckwith J. Tight regulation, modulation, and high-level expression by vectors containing the arabinose PBAD promoter. J Bacteriol. 1995;177(14):4121-30. PubMed PMID: 7608087; PubMed Central PMCID: PMCPMC177145.
2. Pompeo F, van Heijenoort J, Mengin-Lecreulx D. Probing the role of cysteine residues in glucosamine-1-phosphate acetyltransferase activity of the bifunctional GlmU protein from *Escherichia coli*: site-directed mutagenesis and characterization of the mutant enzymes. J Bacteriol. 1998;180(18):4799-803. Epub 1998/09/12. PubMed PMID: 9733680; PubMed Central PMCID: PMCPMC107502.
3. Jolly L, Ferrari P, Blanot D, Van Heijenoort J, Fassy F, Mengin-Lecreulx D. Reaction mechanism of phosphoglucosamine mutase from *Escherichia coli*. European journal of biochemistry. 1999;262(1):202-10. Epub 1999/05/07. PubMed PMID: 10231382.
4. Tavares IM, Jolly L, Pompeo F, Leitao JH, Fialho AM, Sa-Correia I, et al. Identification of the *Pseudomonas aeruginosa glmM* gene, encoding phosphoglucosamine mutase. J Bacteriol. 2000;182(16):4453-7. Epub 2000/07/27. PubMed PMID: 10913078; PubMed Central PMCID: PMCPMC94616.
5. Boles BR, Thoendel M, Roth AJ, Horswill AR. Identification of genes involved in polysaccharide-independent *Staphylococcus aureus* biofilm formation. PLoS One. 2010;5(4):e10146. Epub 2010/04/27. doi: 10.1371/journal.pone.0010146. PubMed PMID: 20418950.
