# Supplementary material for: Inhibition of the Staphylococcus aureus c-di-AMP cyclase DacA by direct interaction with the phosphoglucosamine mutase GlmM
Source: PLoS Pathog. 2019 Jan 22;15(1):e1007537. doi: 10.1371/journal.ppat.1007537 (PMC6368335; doi:10.1371/journal.ppat.1007537)
Supplement: S6 Table — (PDF) [file ppat.1007537.s015.pdf]

S6 Table: Primers used in this study

Number	Name	Sequence
ANG928	F-KpnI-02407	TTTGGTACCTATTACCCGGAGGAGATG
ANG1135	5-NheI-101AA-SAV2163	CTAGCTAGCTTTTTAAAACGCTATACTTCTAATACGTATAG
ANG1137	3-EcoRI-SAV2163	CGGAATTCTTATTTCACACCTTTCTTTTGAAAGCGTG
ANG1244	5-seq-pBAD18	GCACGGCGTCACACTTTGCTATGCC
ANG1245	3-seq-pBAD18	GGCAAATTCTGTTTTATCAGACCGC
ANG2342	5-NcoI-GlmM	CACGCCATGGGAAAATATTTTGGTACAGACGGAGTAAGAGG
ANG2343	3-XhoI-GlmM	CCGCTCGAGGCCGCTGCTGCCGCGCGGCACCAGTTTATCTAATCCCATTTTATCTTGAACC
ANG2475	R_HindIII_dacA	GCGCAAGCTTTTATTTCACACCTTTCTTTTGAAAGCG
ANG2476	R_HindIII_dacAybbr	GCGCAAGCTTTTATTTTACATTTATATAAGCCTTCG
ANG2477	R_HindIII_dacAybbr glmM	GCGCAAGCTTTTATTTATCTAATCCCATTTTATCTTG
ANG2597	front-YbbR-no start	CATTTACTTTCTAGCTAATTATTTCACACCTTTCTTTTGAAAGCG
ANG2598	back-YbbR-no start	GTGAAATAATTAGCTAGAAAGTAAATGGGGCTTG
ANG2616	3-front-dacA-N166K/T172K	GGTTTGTTAGGTATAAAGACTTTAATTAAAAGTTCTTGCG
ANG2617	5-back-dacA-N166K/T172K	ATTAAAGTCTTTATACCTAACAAACCTTTACATGATGGTGC
ANG2618	3-front-dacA-N166C/T172C	GGGCAGTTAGGTATAAAGACGCAAATTAAAAGTTCTTGCGAAATATTTG
ANG2619	5-back-dacA-N166C/T172C	TTTGCGTCTTTATACCTAACTGCCCTTTACATGATGGTGCAATG

Restriction sites in primer sequences are underlined
